# Supplementary material for: Beef cattle that respond differently to fescue toxicosis have distinct gastrointestinal tract microbiota
Source: PLoS One. 2020 Jul 23;15(7):e0229192. doi: 10.1371/journal.pone.0229192 (PMC7377488; doi:10.1371/journal.pone.0229192)
Supplement: S1 Fig — The error bars represent SEM. (PDF) [file pone.0229192.s001.pdf]

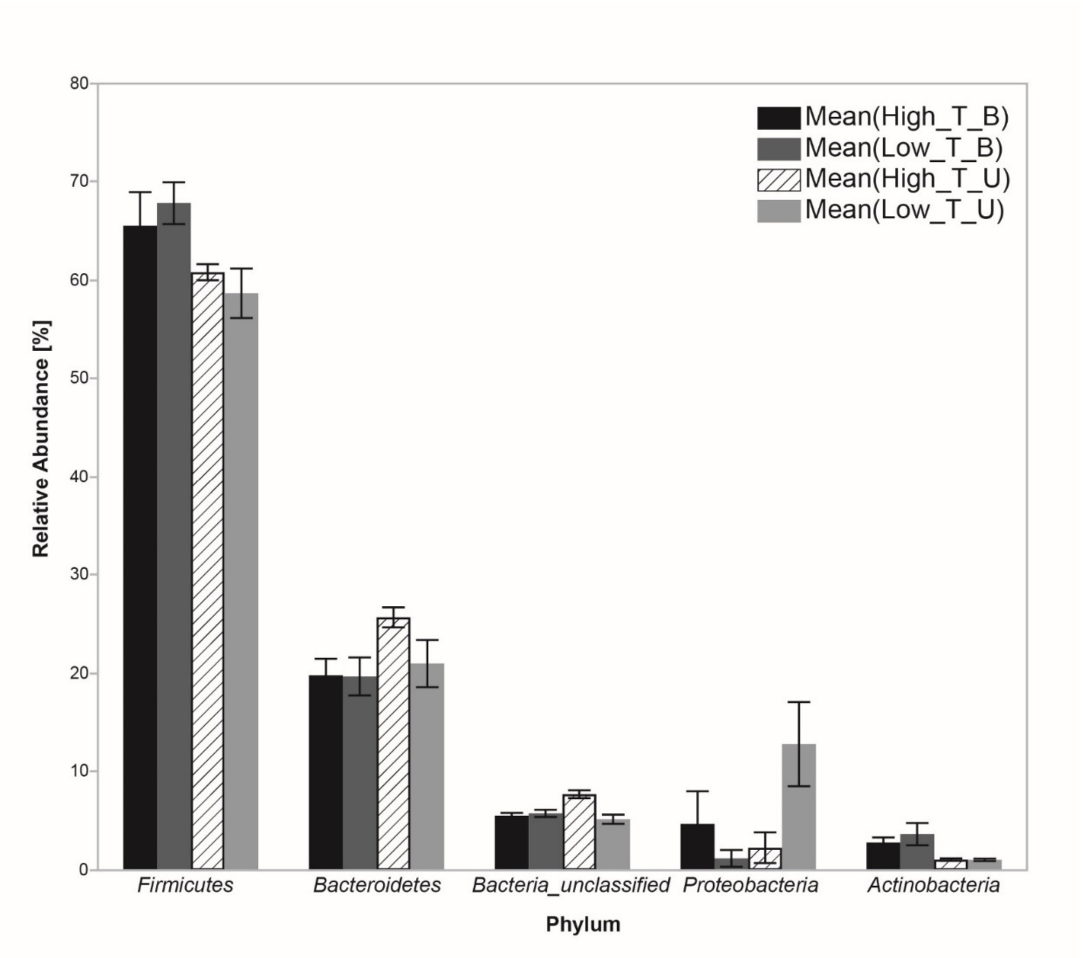

Figure S1: Mean relative abundance of the five most abundant bacterial phyla across all sample sites and groups. The error bars represent SEM.
