# Supplementary material for: Beef cattle that respond differently to fescue toxicosis have distinct gastrointestinal tract microbiota
Source: PLoS One. 2020 Jul 23;15(7):e0229192. doi: 10.1371/journal.pone.0229192 (PMC7377488; doi:10.1371/journal.pone.0229192)
Supplement: S2 Fig — For A, CDA was performed to discriminate animals based on location group (L): BBCFL and UPRS can be found in blue and gold, respectively. For A, CDA was performed to discriminate animals based on tolerance group (T): High (HT) and Low (LT) tolerance to FT can be found in green and red, respectively. For A, the x-axis represents the CS for CAN 1 and y-axis represent the density of the CS data. (PDF) [file pone.0229192.s002.pdf]

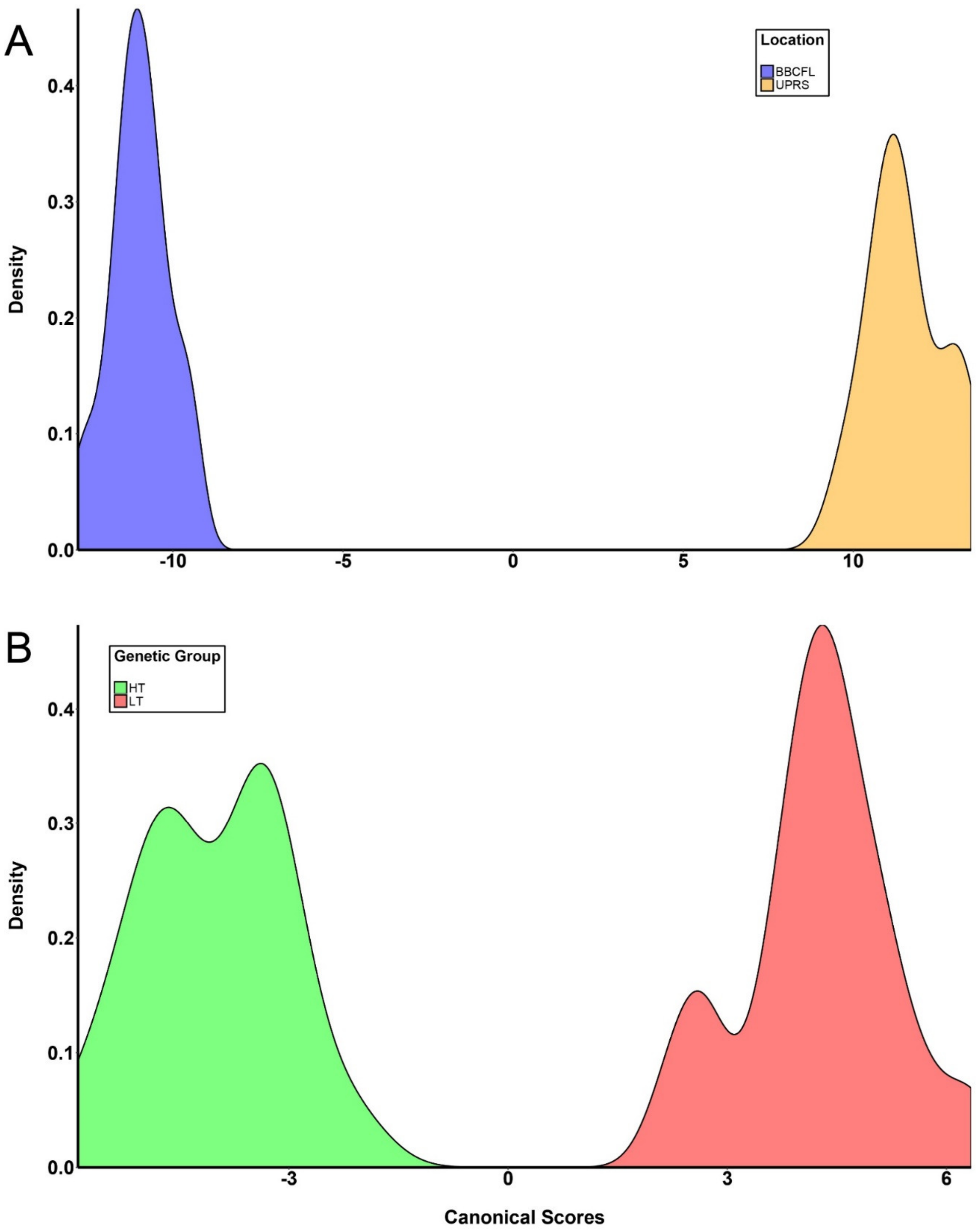

Figure S2: Canonical discriminant analysis (CDA) for response to fescue toxicosis (FT). For A, CDA was performed to discriminate animals based on location group (L): BBCFL and UPRS can be found in blue and gold, respectively. For A, CDA was performed to discriminate animals based on genetic group (G): High (HT) and Low tolerance to FT can be found in green and red, respectively. For A, the x-axis represents the CS for CAN 1 and y-axis represent the density of the CS data.
