# Supplementary material for: Beef cattle that respond differently to fescue toxicosis have distinct gastrointestinal tract microbiota
Source: PLoS One. 2020 Jul 23;15(7):e0229192. doi: 10.1371/journal.pone.0229192 (PMC7377488; doi:10.1371/journal.pone.0229192)
Supplement: S1 Table — Cells shaded grey are statistically significantly (q < 0.05) different for the specific effect. (PDF) [file pone.0229192.s003.pdf]

Table S1: Probability values and adjusted FDR (q-values) values for the 50 most abundant bacterial and fungal OTUs for Location effect, Tolerance effect and the interaction (L*T) between the two effects. Cells shaded grey are statistically significantly (q < 0.05) different for the specific effect

Method	OTU	Location	P-value	q-values		Tolerance	P-value	q-values		Interaction	P-value	q-values
16S	OTU01	L	0.098975	0.216675		T	0.860291	0.740793		L*T	0.302872	0.208735
16S	OTU02	L	0.11427	0.243576		T	0.219462	0.388454		L*T	0.066066	0.117503
16S	OTU03	L	0.022894	0.06623		T	1.60E-05	0.000127		L*T	0.321515	0.216902
16S	OTU04	L	0.26953	0.507719		T	0.405114	0.533222		L*T	0.232149	0.185378
16S	OTU05	L	0.140452	0.284416		T	0.515098	0.623484		L*T	0.252217	0.189784
16S	OTU06	L	0.749982	0.905251		T	0.379597	0.531536		L*T	0.01195	0.031171
16S	OTU07	L	0.051852	0.135484		T	0.971162	0.763994		L*T	0.547352	0.334638
16S	OTU08	L	0.899786	0.991741		T	0.963404	0.763994		L*T	0.285274	0.204206
16S	OTU09	L	0.001529	0.008318		T	0.061462	0.145054		L*T	0.083259	0.124869
16S	OTU10	L	0.00119	0.008035		T	0.194128	0.353429		L*T	0.153796	0.158362
16S	OTU11	L	0.799462	0.925092		T	0.383714	0.531536		L*T	0.048845	0.09101
16S	OTU12	L	0.975449	0.991741		T	0.510285	0.623484		L*T	0.910453	0.463655
16S	OTU13	L	0.316126	0.540612		T	0.575249	0.637349		L*T	0.274168	0.202409
16S	OTU14	L	0.457246	0.698809		T	0.171823	0.324899		L*T	0.169697	0.158474
16S	OTU15	L	0.457064	0.698809		T	0.536322	0.63287		L*T	0.000623	0.004065
16S	OTU16	L	0.823331	0.939293		T	0.762848	0.68464		L*T	0.58848	0.354248
16S	OTU17	L	0.525778	0.744878		T	0.891622	0.757535		L*T	0.110629	0.134785
16S	OTU18	L	0.574342	0.764101		T	0.929977	0.763994		L*T	0.017177	0.039535
16S	OTU19	L	0.003465	0.015188		T	0.407167	0.533222		L*T	0.006682	0.023774
16S	OTU20	L	0.507666	0.744878		T	0.001047	0.004767		L*T	0.00118	0.006593
16S	OTU21	L	0.004944	0.020022		T	0.251621	0.400839		L*T	0.944001	0.463655
16S	OTU22	L	0.796012	0.925092		T	0.436583	0.55639		L*T	0.17422	0.158474
16S	OTU23	L	0.411549	0.66671		T	0.056848	0.139323		L*T	0.074127	0.118063
16S	OTU24	L	0.942812	0.991741		T	0.055112	0.139323		L*T	0.28704	0.204206
16S	OTU25	L	0.001751	0.008863		T	0.832162	0.726387		L*T	0.093405	0.126026
16S	OTU26	L	0.071382	0.175211		T	0.619014	0.637349		L*T	0.009107	0.025453
16S	OTU27	L	0.472442	0.708664		T	0.017104	0.04954		L*T	0.007899	0.023774
16S	OTU28	L	0.970189	0.991741		T	0.620209	0.637349		L*T	0.174612	0.158474
16S	OTU29	L	0.024488	0.068396		T	0.297179	0.440385		L*T	0.454584	0.286887
16S	OTU30	L	0.148468	0.293315		T	0.76127	0.68464		L*T	0.163047	0.158474
16S	OTU31	L	0.584868	0.764101		T	0.605779	0.637349		L*T	0.304076	0.208735
16S	OTU32	L	0.53337	0.744878		T	0.816881	0.722951		L*T	0.139428	0.147448
16S	OTU33	L	0.021649	0.064946		T	0.641273	0.637349		L*T	0.071071	0.118063
16S	OTU34	L	0.212998	0.410782		T	0.574674	0.637349		L*T	0.104068	0.134785
16S	OTU35	L	0.686634	0.842687		T	0.960423	0.763994		L*T	0.837595	0.438688
16S	OTU36	L	1.23E-06	1.99E-05		T	0.640607	0.637349		L*T	0.241885	0.187424
16S	OTU37	L	0.579914	0.764101		T	0.734437	0.68464		L*T	0.184782	0.158474
16S	OTU38	L	0.759964	0.905251		T	0.019128	0.052995		L*T	0.007263	0.023774

16S	OTU39	L	0.529594	0.744878		T	0.288594	0.440385		L*T	0.170991	0.158474
16S	OTU40	L	0.401337	0.663435		T	0.246879	0.400839		L*T	0.766986	0.416816
16S	OTU41	L	0.453218	0.698809		T	0.140312	0.2794		L*T	0.196463	0.160151
16S	OTU42	L	0.954269	0.991741		T	0.752793	0.68464		L*T	0.086165	0.124869
16S	OTU43	L	0.000126	0.001452		T	0.247813	0.400839		L*T	0.091452	0.126026
16S	OTU44	L	0.312268	0.540612		T	0.518583	0.623484		L*T	0.184449	0.158474
16S	OTU45	L	0.003236	0.015188		T	0.574436	0.637349		L*T	0.003687	0.016029
16S	OTU46	L	0.892628	0.991741		T	0.29324	0.440385		L*T	0.696756	0.389468
16S	OTU47	L	0.016355	0.051988		T	0.592498	0.637349		L*T	0.928212	0.463655
16S	OTU48	L	0.016688	0.051988		T	0.410035	0.533222		L*T	0.840868	0.438688
16S	OTU49	L	0.320363	0.540612		T	0.926112	0.763994		L*T	0.624912	0.363909
16S	OTU50	L	0.001459	0.008318		T	0.757494	0.68464		L*T	0.113675	0.134785
ITS	OTU01	L	1.56E-07	4.20E-06		T	0.000995	0.004767		L*T	0.190356	0.158474
ITS	OTU02	L	0.555138	0.762138		T	1.23E-09	2.61E-08		L*T	0.190321	0.158474
ITS	OTU03	L	0.011638	0.040985		T	3.60E-05	0.000229		L*T	0.075434	0.118063
ITS	OTU04	L	0.014225	0.04801		T	5.46E-11	1.74E-09		L*T	2.28E-05	0.000298
ITS	OTU05	L	9.32E-12	7.55E-10		T	2.35E-08	2.99E-07		L*T	0.007509	0.023774
ITS	OTU06	L	0.005561	0.021448		T	2.38E-13	1.52E-11		L*T	1.51E-06	5.90E-05
ITS	OTU07	L	2.19E-07	4.43E-06		T	0.238885	0.400839		L*T	0.452412	0.286887
ITS	OTU08	L	0.003563	0.015188		T	0.332126	0.480987		L*T	5.25E-06	0.000103
ITS	OTU10	L	0.067966	0.172038		T	0.001812	0.007699		L*T	0.021149	0.045974
ITS	OTU11	L	0.318137	0.540612		T	0.650141	0.637349		L*T	0.24429	0.187424
ITS	OTU12	L	7.78E-10	3.15E-08		T	8.66E-09	1.38E-07		L*T	0.001711	0.00837
ITS	OTU13	L	0.962469	0.991741		T	0.016418	0.04954		L*T	0.108505	0.134785
ITS	OTU14	L	0.011027	0.0406		T	0.002687	0.010701		L*T	0.60605	0.359297
ITS	OTU15	L	0.306404	0.540612		T	0.087452	0.185752		L*T	5.69E-05	0.000557
ITS	OTU16	L	0.990622	0.991741		T	0.003626	0.013591		L*T	0.000121	0.00095
ITS	OTU17	L	0.000567	0.004176		T	2.87E-05	0.000203		L*T	0.024222	0.049883
ITS	OTU19	L	0.68386	0.842687		T	0.637607	0.637349		L*T	0.998959	0.482561
ITS	OTU20	L	0.956295	0.991741		T	0.000952	0.004767		L*T	0.63243	0.363909
ITS	OTU21	L	0.133129	0.276498		T	0.173358	0.324899		L*T	0.137253	0.147448
ITS	OTU22	L	0.084967	0.196638		T	0.016191	0.04954		L*T	0.747654	0.412033
ITS	OTU24	L	0.092698	0.20857		T	0.021663	0.057515		L*T	0.368463	0.244361
ITS	OTU25	L	1.24E-05	0.000167		T	0.071638	0.157408		L*T	0.947973	0.463655
ITS	OTU26	L	0.000327	0.002771		T	0.004185	0.014815		L*T	0.831647	0.438688
ITS	OTU27	L	0.037	0.099899		T	0.000299	0.00173		L*T	0.014022	0.034291
ITS	OTU31	L	0.596563	0.76701		T	0.011809	0.039603		L*T	0.491424	0.305214
ITS	OTU34	L	0.000185	0.001876		T	0.961313	0.763994		L*T	0.383905	0.250358
ITS	OTU36	L	0.991741	0.991741		T	0.136528	0.2794		L*T	0.946189	0.463655
ITS	OTU38	L	0.000342	0.002771		T	6.06E-06	5.52E-05		L*T	0.035328	0.069115
ITS	OTU42	L	0.658321	0.833187		T	0.75984	0.68464		L*T	0.671263	0.380656
ITS	OTU44	L	0.078933	0.188047		T	0.063921	0.145469		L*T	0.118605	0.136494
ITS	OTU49	L	0.00154	0.008318		T	4.87E-07	5.17E-06		L*T	0.123355	0.137904
