# Supplementary material for: Beef cattle that respond differently to fescue toxicosis have distinct gastrointestinal tract microbiota
Source: PLoS One. 2020 Jul 23;15(7):e0229192. doi: 10.1371/journal.pone.0229192 (PMC7377488; doi:10.1371/journal.pone.0229192)
Supplement: S2 Table — Fungal OTUs highlighted in grey demonstrate significant interaction between Tolerance and Location but share directionality of the Location effect as noted in the manuscript and in Fig 3. (PDF) [file pone.0229192.s004.pdf]

Table S2: Log2 Fold Change values for the 50 most abundant bacterial and fungal OTUs for all combinations of Location, Tolerance and their interactions (T*L). Fungal OTUs highlighted in grey demonstrate significant interaction between Tolerance and Location but share directionality of the Location effect as noted in the manuscript and Fig. 3.

Method	OTU	Effect	Location 1	Location 2	Tolerance Group 1	Tolerance Group 2	log2FC	Lower 95p CI	Upper 95p CI
16S	OTU01	L	Butner	UPRS			-0.23255	-0.51132	0.046214
16S	OTU01	T			High T	Low T	-0.02013	-0.25119	0.210935
16S	OTU01	L*T	Butner	Butner	High T	Low T	0.122171	-0.25625	0.500597
16S	OTU01	L*T	Butner	UPRS	High T	High T	-0.09025	-0.41826	0.237756
16S	OTU01	L*T	Butner	UPRS	High T	Low T	-0.25268	-0.59125	0.085896
16S	OTU01	L*T	Butner	UPRS	Low T	High T	-0.21243	-0.59657	0.171719
16S	OTU01	L*T	Butner	UPRS	Low T	Low T	-0.37485	-0.82325	0.073552
16S	OTU01	L*T	UPRS	UPRS	High T	Low T	-0.16242	-0.5042	0.179347
16S	OTU02	L	Butner	UPRS			1.155745	-0.29422	2.605707
16S	OTU02	T			High T	Low T	-0.85266	-2.23933	0.534008
16S	OTU02	L*T	Butner	Butner	High T	Low T	-2.72997	-5.56084	0.100895
16S	OTU02	L*T	Butner	UPRS	High T	High T	-0.72157	-3.04253	1.599394
16S	OTU02	L*T	Butner	UPRS	High T	Low T	0.303086	-1.72776	2.333929
16S	OTU02	L*T	Butner	UPRS	Low T	High T	2.008404	0.026955	3.989854
16S	OTU02	L*T	Butner	UPRS	Low T	Low T	3.033059	0.407538	5.658581
16S	OTU02	L*T	UPRS	UPRS	High T	Low T	1.024655	-0.95204	3.001353
16S	OTU03	L	Butner	UPRS			-2.48604	-4.60461	-0.36748
16S	OTU03	T			High T	Low T	4.387014	2.62636	6.147668
16S	OTU03	L*T	Butner	Butner	High T	Low T	5.526475	2.741633	8.311316
16S	OTU03	L*T	Butner	UPRS	High T	High T	-1.34658	-3.92309	1.229924
16S	OTU03	L*T	Butner	UPRS	High T	Low T	1.900971	-0.87891	4.680856
16S	OTU03	L*T	Butner	UPRS	Low T	High T	-6.87306	-9.60228	-4.14383
16S	OTU03	L*T	Butner	UPRS	Low T	Low T	-3.6255	-7.2261	-0.02491
16S	OTU03	L*T	UPRS	UPRS	High T	Low T	3.247553	0.235827	6.259279
16S	OTU04	L	Butner	UPRS			1.166088	-0.94782	3.279993
16S	OTU04	T			High T	Low T	-0.72757	-2.48419	1.029047
16S	OTU04	L*T	Butner	Butner	High T	Low T	-1.88246	-4.79518	1.030253
16S	OTU04	L*T	Butner	UPRS	High T	High T	0.011197	-2.32155	2.343945
16S	OTU04	L*T	Butner	UPRS	High T	Low T	0.438517	-1.97276	2.849796
16S	OTU04	L*T	Butner	UPRS	Low T	High T	1.893658	-1.155	4.94232
16S	OTU04	L*T	Butner	UPRS	Low T	Low T	2.320978	-0.98911	5.631065
16S	OTU04	L*T	UPRS	UPRS	High T	Low T	0.42732	-1.84176	2.696396
16S	OTU05	L	Butner	UPRS			-0.19505	-0.45789	0.067789
16S	OTU05	T			High T	Low T	0.070643	-0.14797	0.289253
16S	OTU05	L*T	Butner	Butner	High T	Low T	0.219277	-0.13812	0.576675
16S	OTU05	L*T	Butner	UPRS	High T	High T	-0.04642	-0.35491	0.262079

16S	OTU05	L*T	Butner	UPRS	High T	Low T	-0.12441	-0.44294	0.194127
16S	OTU05	L*T	Butner	UPRS	Low T	High T	-0.26569	-0.6294	0.098017
16S	OTU05	L*T	Butner	UPRS	Low T	Low T	-0.34368	-0.76538	0.078016
16S	OTU05	L*T	UPRS	UPRS	High T	Low T	-0.07799	-0.39842	0.242445
16S	OTU06	L	Butner	UPRS			0.09357	-0.49941	0.686548
16S	OTU06	T			High T	Low T	0.209729	-0.26975	0.689212
16S	OTU06	L*T	Butner	Butner	High T	Low T	0.951984	0.182552	1.721416
16S	OTU06	L*T	Butner	UPRS	High T	High T	0.835825	0.162048	1.509601
16S	OTU06	L*T	Butner	UPRS	High T	Low T	0.303299	-0.41083	1.017423
16S	OTU06	L*T	Butner	UPRS	Low T	High T	-0.11616	-0.92429	0.691976
16S	OTU06	L*T	Butner	UPRS	Low T	Low T	-0.64868	-1.59355	0.296185
16S	OTU06	L*T	UPRS	UPRS	High T	Low T	-0.53253	-1.24757	0.182519
16S	OTU07	L	Butner	UPRS			-0.39483	-0.79302	0.003364
16S	OTU07	T			High T	Low T	-0.006	-0.3415	0.329502
16S	OTU07	L*T	Butner	Butner	High T	Low T	0.113895	-0.43131	0.659103
16S	OTU07	L*T	Butner	UPRS	High T	High T	-0.27493	-0.77002	0.220153
16S	OTU07	L*T	Butner	UPRS	High T	Low T	-0.40083	-0.90261	0.100943
16S	OTU07	L*T	Butner	UPRS	Low T	High T	-0.38883	-0.92778	0.150118
16S	OTU07	L*T	Butner	UPRS	Low T	Low T	-0.51473	-1.14281	0.11336
16S	OTU07	L*T	UPRS	UPRS	High T	Low T	-0.1259	-0.62626	0.374463
16S	OTU08	L	Butner	UPRS			0.024388	-0.36698	0.415753
16S	OTU08	T			High T	Low T	-0.00753	-0.3393	0.324239
16S	OTU08	L*T	Butner	Butner	High T	Low T	0.205887	-0.33742	0.749197
16S	OTU08	L*T	Butner	UPRS	High T	High T	0.237807	-0.24402	0.719634
16S	OTU08	L*T	Butner	UPRS	High T	Low T	0.016857	-0.46756	0.501272
16S	OTU08	L*T	Butner	UPRS	Low T	High T	0.03192	-0.50828	0.572121
16S	OTU08	L*T	Butner	UPRS	Low T	Low T	-0.18903	-0.8169	0.438835
16S	OTU08	L*T	UPRS	UPRS	High T	Low T	-0.22095	-0.71592	0.274022
16S	OTU09	L	Butner	UPRS			-1.93005	-3.06453	-0.79556
16S	OTU09	T			High T	Low T	0.895418	-0.04561	1.836443
16S	OTU09	L*T	Butner	Butner	High T	Low T	1.898654	0.30772	3.489588
16S	OTU09	L*T	Butner	UPRS	High T	High T	-0.92681	-2.19714	0.343522
16S	OTU09	L*T	Butner	UPRS	High T	Low T	-1.03463	-2.3556	0.286345
16S	OTU09	L*T	Butner	UPRS	Low T	High T	-2.82546	-4.43798	-1.21294
16S	OTU09	L*T	Butner	UPRS	Low T	Low T	-2.93328	-4.82353	-1.04303
16S	OTU09	L*T	UPRS	UPRS	High T	Low T	-0.10782	-1.46893	1.253298
16S	OTU10	L	Butner	UPRS			-0.65787	-1.0345	-0.28123
16S	OTU10	T			High T	Low T	-0.20439	-0.51829	0.10951
16S	OTU10	L*T	Butner	Butner	High T	Low T	0.072358	-0.45501	0.599726
16S	OTU10	L*T	Butner	UPRS	High T	High T	-0.38112	-0.82937	0.067125
16S	OTU10	L*T	Butner	UPRS	High T	Low T	-0.86226	-1.31758	-0.40693

16S	OTU10	L*T	Butner	UPRS	Low T	High T	-0.45348	-0.97641	0.069446
16S	OTU10	L*T	Butner	UPRS	Low T	Low T	-0.93461	-1.55152	-0.31771
16S	OTU10	L*T	UPRS	UPRS	High T	Low T	-0.48113	-0.94668	-0.01558
16S	OTU11	L	Butner	UPRS			0.092835	-0.64535	0.831021
16S	OTU11	T			High T	Low T	0.252713	-0.33012	0.835547
16S	OTU11	L*T	Butner	Butner	High T	Low T	0.951297	0.01187	1.890723
16S	OTU11	L*T	Butner	UPRS	High T	High T	0.791418	-0.02976	1.612593
16S	OTU11	L*T	Butner	UPRS	High T	Low T	0.345548	-0.53828	1.22938
16S	OTU11	L*T	Butner	UPRS	Low T	High T	-0.15988	-1.15389	0.834137
16S	OTU11	L*T	Butner	UPRS	Low T	Low T	-0.60575	-1.78094	0.569441
16S	OTU11	L*T	UPRS	UPRS	High T	Low T	-0.44587	-1.31904	0.427303
16S	OTU12	L	Butner	UPRS			0.004225	-0.27324	0.281687
16S	OTU12	T			High T	Low T	-0.07708	-0.3129	0.158732
16S	OTU12	L*T	Butner	Butner	High T	Low T	-0.06182	-0.44258	0.318935
16S	OTU12	L*T	Butner	UPRS	High T	High T	0.019486	-0.31625	0.355224
16S	OTU12	L*T	Butner	UPRS	High T	Low T	-0.07286	-0.41361	0.267896
16S	OTU12	L*T	Butner	UPRS	Low T	High T	0.081309	-0.30479	0.467412
16S	OTU12	L*T	Butner	UPRS	Low T	Low T	-0.01104	-0.44881	0.426742
16S	OTU12	L*T	UPRS	UPRS	High T	Low T	-0.09235	-0.43387	0.249181
16S	OTU13	L	Butner	UPRS			-0.19235	-0.57706	0.19237
16S	OTU13	T			High T	Low T	-0.09045	-0.41587	0.234979
16S	OTU13	L*T	Butner	Butner	High T	Low T	0.123731	-0.41053	0.65799
16S	OTU13	L*T	Butner	UPRS	High T	High T	0.021834	-0.4501	0.493765
16S	OTU13	L*T	Butner	UPRS	High T	Low T	-0.28279	-0.7578	0.192213
16S	OTU13	L*T	Butner	UPRS	Low T	High T	-0.1019	-0.63311	0.429314
16S	OTU13	L*T	Butner	UPRS	Low T	Low T	-0.40652	-1.02361	0.210567
16S	OTU13	L*T	UPRS	UPRS	High T	Low T	-0.30463	-0.78824	0.178985
16S	OTU14	L	Butner	UPRS			-0.3959	-1.46756	0.675752
16S	OTU14	T			High T	Low T	0.647867	-0.29631	1.592044
16S	OTU14	L*T	Butner	Butner	High T	Low T	1.439546	-0.20569	3.084779
16S	OTU14	L*T	Butner	UPRS	High T	High T	0.395775	-0.83897	1.630522
16S	OTU14	L*T	Butner	UPRS	High T	Low T	0.251963	-0.97706	1.480988
16S	OTU14	L*T	Butner	UPRS	Low T	High T	-1.04377	-2.64668	0.559142
16S	OTU14	L*T	Butner	UPRS	Low T	Low T	-1.18758	-3.03353	0.658364
16S	OTU14	L*T	UPRS	UPRS	High T	Low T	-0.14381	-1.45201	1.164384
16S	OTU15	L	Butner	UPRS			0.244219	-0.41658	0.905015
16S	OTU15	T			High T	Low T	0.16475	-0.37208	0.701578
16S	OTU15	L*T	Butner	Butner	High T	Low T	1.325702	0.463959	2.187445
16S	OTU15	L*T	Butner	UPRS	High T	High T	1.405171	0.671946	2.138396
16S	OTU15	L*T	Butner	UPRS	High T	Low T	0.408969	-0.37296	1.190898
16S	OTU15	L*T	Butner	UPRS	Low T	High T	0.079469	-0.8361	0.995034

16S	OTU15	L*T	Butner	UPRS	Low T	Low T	-0.91673	-1.97168	0.138208
16S	OTU15	L*T	UPRS	UPRS	High T	Low T	-0.9962	-1.77786	-0.21455
16S	OTU16	L	Butner	UPRS			-0.04681	-0.4704	0.376779
16S	OTU16	T			High T	Low T	0.053262	-0.30323	0.409755
16S	OTU16	L*T	Butner	Butner	High T	Low T	0.169155	-0.42328	0.761586
16S	OTU16	L*T	Butner	UPRS	High T	High T	0.069082	-0.43633	0.574493
16S	OTU16	L*T	Butner	UPRS	High T	Low T	0.006451	-0.50794	0.520845
16S	OTU16	L*T	Butner	UPRS	Low T	High T	-0.10007	-0.69035	0.490207
16S	OTU16	L*T	Butner	UPRS	Low T	Low T	-0.1627	-0.85302	0.527608
16S	OTU16	L*T	UPRS	UPRS	High T	Low T	-0.06263	-0.58829	0.463027
16S	OTU17	L	Butner	UPRS			-0.12937	-0.54018	0.281433
16S	OTU17	T			High T	Low T	-0.02334	-0.36956	0.322875
16S	OTU17	L*T	Butner	Butner	High T	Low T	-0.35504	-0.91999	0.209915
16S	OTU17	L*T	Butner	UPRS	High T	High T	-0.46107	-0.96109	0.038949
16S	OTU17	L*T	Butner	UPRS	High T	Low T	-0.15272	-0.66277	0.357332
16S	OTU17	L*T	Butner	UPRS	Low T	High T	-0.10603	-0.66915	0.457094
16S	OTU17	L*T	Butner	UPRS	Low T	Low T	0.202322	-0.45084	0.855481
16S	OTU17	L*T	UPRS	UPRS	High T	Low T	0.308352	-0.20123	0.817933
16S	OTU18	L	Butner	UPRS			0.132521	-0.34315	0.608191
16S	OTU18	T			High T	Low T	0.017369	-0.3821	0.416836
16S	OTU18	L*T	Butner	Butner	High T	Low T	0.60383	-0.04482	1.25248
16S	OTU18	L*T	Butner	UPRS	High T	High T	0.718982	0.157783	1.280181
16S	OTU18	L*T	Butner	UPRS	High T	Low T	0.149891	-0.42733	0.727108
16S	OTU18	L*T	Butner	UPRS	Low T	High T	0.115152	-0.54703	0.777339
16S	OTU18	L*T	Butner	UPRS	Low T	Low T	-0.45394	-1.22159	0.313708
16S	OTU18	L*T	UPRS	UPRS	High T	Low T	-0.56909	-1.16078	0.022602
16S	OTU19	L	Butner	UPRS			-0.47537	-0.78209	-0.16865
16S	OTU19	T			High T	Low T	-0.10639	-0.36438	0.151607
16S	OTU19	L*T	Butner	Butner	High T	Low T	0.338587	-0.08529	0.762462
16S	OTU19	L*T	Butner	UPRS	High T	High T	-0.0304	-0.4136	0.352806
16S	OTU19	L*T	Butner	UPRS	High T	Low T	-0.58176	-0.96698	-0.19654
16S	OTU19	L*T	Butner	UPRS	Low T	High T	-0.36898	-0.78478	0.046808
16S	OTU19	L*T	Butner	UPRS	Low T	Low T	-0.92035	-1.40674	-0.43395
16S	OTU19	L*T	UPRS	UPRS	High T	Low T	-0.55136	-0.93701	-0.16571
16S	OTU20	L	Butner	UPRS			0.161594	-0.32969	0.652873
16S	OTU20	T			High T	Low T	0.726864	0.316149	1.137579
16S	OTU20	L*T	Butner	Butner	High T	Low T	1.582721	0.924732	2.24071
16S	OTU20	L*T	Butner	UPRS	High T	High T	1.017451	0.440954	1.593948
16S	OTU20	L*T	Butner	UPRS	High T	Low T	0.888458	0.283044	1.493872
16S	OTU20	L*T	Butner	UPRS	Low T	High T	-0.56527	-1.23874	0.108196
16S	OTU20	L*T	Butner	UPRS	Low T	Low T	-0.69426	-1.48776	0.09923

16S	OTU20	L*T	UPRS	UPRS	High T	Low T	-0.12899	-0.74842	0.490438
16S	OTU21	L	Butner	UPRS			0.595246	0.193681	0.99681
16S	OTU21	T			High T	Low T	-0.19404	-0.53257	0.14449
16S	OTU21	L*T	Butner	Butner	High T	Low T	-0.20802	-0.76056	0.344532
16S	OTU21	L*T	Butner	UPRS	High T	High T	0.581269	0.098802	1.063735
16S	OTU21	L*T	Butner	UPRS	High T	Low T	0.401207	-0.08941	0.891826
16S	OTU21	L*T	Butner	UPRS	Low T	High T	0.789284	0.231606	1.346962
16S	OTU21	L*T	Butner	UPRS	Low T	Low T	0.609223	-0.03356	1.252007
16S	OTU21	L*T	UPRS	UPRS	High T	Low T	-0.18006	-0.67739	0.317263
16S	OTU22	L	Butner	UPRS			0.253488	-1.72727	2.234248
16S	OTU22	T			High T	Low T	-0.65043	-2.33208	1.03122
16S	OTU22	L*T	Butner	Butner	High T	Low T	-2.05192	-4.93724	0.833394
16S	OTU22	L*T	Butner	UPRS	High T	High T	-1.148	-3.65745	1.36144
16S	OTU22	L*T	Butner	UPRS	High T	Low T	-0.39694	-2.954	2.160109
16S	OTU22	L*T	Butner	UPRS	Low T	High T	0.90392	-1.73506	3.542897
16S	OTU22	L*T	Butner	UPRS	Low T	Low T	1.65498	-1.50562	4.815577
16S	OTU22	L*T	UPRS	UPRS	High T	Low T	0.75106	-1.65117	3.15329
16S	OTU23	L	Butner	UPRS			0.175683	-0.25441	0.605774
16S	OTU23	T			High T	Low T	-0.36426	-0.73978	0.011262
16S	OTU23	L*T	Butner	Butner	High T	Low T	-0.75059	-1.35765	-0.14352
16S	OTU23	L*T	Butner	UPRS	High T	High T	-0.21064	-0.74549	0.3242
16S	OTU23	L*T	Butner	UPRS	High T	Low T	-0.18858	-0.72825	0.351099
16S	OTU23	L*T	Butner	UPRS	Low T	High T	0.539943	-0.06067	1.140558
16S	OTU23	L*T	Butner	UPRS	Low T	Low T	0.56201	-0.10676	1.230776
16S	OTU23	L*T	UPRS	UPRS	High T	Low T	0.022067	-0.5041	0.548238
16S	OTU24	L	Butner	UPRS			-0.01012	-0.29534	0.27509
16S	OTU24	T			High T	Low T	-0.23008	-0.46551	0.005344
16S	OTU24	L*T	Butner	Butner	High T	Low T	-0.38197	-0.76595	0.002005
16S	OTU24	L*T	Butner	UPRS	High T	High T	-0.16202	-0.50671	0.182677
16S	OTU24	L*T	Butner	UPRS	High T	Low T	-0.24021	-0.59397	0.113554
16S	OTU24	L*T	Butner	UPRS	Low T	High T	0.219959	-0.16527	0.605184
16S	OTU24	L*T	Butner	UPRS	Low T	Low T	0.141767	-0.31343	0.596963
16S	OTU24	L*T	UPRS	UPRS	High T	Low T	-0.07819	-0.43419	0.277806
16S	OTU25	L	Butner	UPRS			2.994735	1.2085	4.780969
16S	OTU25	T			High T	Low T	0.162786	-1.38908	1.714657
16S	OTU25	L*T	Butner	Butner	High T	Low T	1.536668	-1.03913	4.112468
16S	OTU25	L*T	Butner	UPRS	High T	High T	4.368617	2.590368	6.146866
16S	OTU25	L*T	Butner	UPRS	High T	Low T	3.157521	1.225334	5.089708
16S	OTU25	L*T	Butner	UPRS	Low T	High T	2.831949	0.09982	5.564077
16S	OTU25	L*T	Butner	UPRS	Low T	Low T	1.620853	-1.28728	4.52898
16S	OTU25	L*T	UPRS	UPRS	High T	Low T	-1.2111	-3.06041	0.638221

16S	OTU26	L	Butner	UPRS			-0.81935	-1.71426	0.075561
16S	OTU26	T			High T	Low T	0.18204	-0.55642	0.920503
16S	OTU26	L*T	Butner	Butner	High T	Low T	1.428316	0.241023	2.615608
16S	OTU26	L*T	Butner	UPRS	High T	High T	0.426928	-0.63108	1.484937
16S	OTU26	L*T	Butner	UPRS	High T	Low T	-0.63731	-1.74642	0.471805
16S	OTU26	L*T	Butner	UPRS	Low T	High T	-1.00139	-2.21062	0.207847
16S	OTU26	L*T	Butner	UPRS	Low T	Low T	-2.06562	-3.53337	-0.59788
16S	OTU26	L*T	UPRS	UPRS	High T	Low T	-1.06423	-2.22739	0.09892
16S	OTU27	L	Butner	UPRS			-0.39808	-1.51326	0.717099
16S	OTU27	T			High T	Low T	-1.28506	-2.32566	-0.24446
16S	OTU27	L*T	Butner	Butner	High T	Low T	-2.87106	-4.569	-1.17312
16S	OTU27	L*T	Butner	UPRS	High T	High T	-1.98408	-3.32103	-0.64714
16S	OTU27	L*T	Butner	UPRS	High T	Low T	-1.68314	-3.04704	-0.31923
16S	OTU27	L*T	Butner	UPRS	Low T	High T	0.88698	-0.78416	2.55812
16S	OTU27	L*T	Butner	UPRS	Low T	Low T	1.187925	-0.62838	3.004234
16S	OTU27	L*T	UPRS	UPRS	High T	Low T	0.300945	-1.07086	1.672749
16S	OTU28	L	Butner	UPRS			0.00756	-0.40131	0.416429
16S	OTU28	T			High T	Low T	0.085358	-0.26209	0.43281
16S	OTU28	L*T	Butner	Butner	High T	Low T	0.373802	-0.19853	0.946131
16S	OTU28	L*T	Butner	UPRS	High T	High T	0.296004	-0.21449	0.806496
16S	OTU28	L*T	Butner	UPRS	High T	Low T	0.092919	-0.41655	0.602383
16S	OTU28	L*T	Butner	UPRS	Low T	High T	-0.0778	-0.64015	0.484554
16S	OTU28	L*T	Butner	UPRS	Low T	Low T	-0.28088	-0.93809	0.376321
16S	OTU28	L*T	UPRS	UPRS	High T	Low T	-0.20309	-0.72468	0.318505
16S	OTU29	L	Butner	UPRS			-0.37172	-0.69242	-0.05101
16S	OTU29	T			High T	Low T	-0.13976	-0.40838	0.128861
16S	OTU29	L*T	Butner	Butner	High T	Low T	-0.02302	-0.45844	0.4124
16S	OTU29	L*T	Butner	UPRS	High T	High T	-0.25498	-0.63727	0.127312
16S	OTU29	L*T	Butner	UPRS	High T	Low T	-0.51147	-0.9034	-0.11955
16S	OTU29	L*T	Butner	UPRS	Low T	High T	-0.23196	-0.67514	0.211228
16S	OTU29	L*T	Butner	UPRS	Low T	Low T	-0.48845	-0.9953	0.018394
16S	OTU29	L*T	UPRS	UPRS	High T	Low T	-0.25649	-0.64644	0.133447
16S	OTU30	L	Butner	UPRS			-0.25487	-0.60548	0.095745
16S	OTU30	T			High T	Low T	0.04452	-0.25143	0.340468
16S	OTU30	L*T	Butner	Butner	High T	Low T	0.289933	-0.18995	0.769815
16S	OTU30	L*T	Butner	UPRS	High T	High T	-0.00946	-0.44157	0.422657
16S	OTU30	L*T	Butner	UPRS	High T	Low T	-0.21035	-0.64865	0.227949
16S	OTU30	L*T	Butner	UPRS	Low T	High T	-0.29939	-0.77785	0.179072
16S	OTU30	L*T	Butner	UPRS	Low T	Low T	-0.50028	-1.05192	0.051359
16S	OTU30	L*T	UPRS	UPRS	High T	Low T	-0.20089	-0.63684	0.235058
16S	OTU31	L	Butner	UPRS			0.470895	-1.26715	2.208938

16S	OTU31	T			High T	Low T	0.364243	-1.05913	1.787618
16S	OTU31	L*T	Butner	Butner	High T	Low T	1.29794	-1.12189	3.717768
16S	OTU31	L*T	Butner	UPRS	High T	High T	1.404593	-0.58442	3.393604
16S	OTU31	L*T	Butner	UPRS	High T	Low T	0.835138	-1.23265	2.902926
16S	OTU31	L*T	Butner	UPRS	Low T	High T	0.106652	-2.30536	2.518669
16S	OTU31	L*T	Butner	UPRS	Low T	Low T	-0.4628	-3.41513	2.489531
16S	OTU31	L*T	UPRS	UPRS	High T	Low T	-0.56945	-2.76656	1.627653
16S	OTU32	L	Butner	UPRS			-0.13764	-0.58288	0.307599
16S	OTU32	T			High T	Low T	0.042631	-0.3293	0.414567
16S	OTU32	L*T	Butner	Butner	High T	Low T	0.365279	-0.23176	0.962317
16S	OTU32	L*T	Butner	UPRS	High T	High T	0.185009	-0.34183	0.711844
16S	OTU32	L*T	Butner	UPRS	High T	Low T	-0.09501	-0.63714	0.44713
16S	OTU32	L*T	Butner	UPRS	Low T	High T	-0.18027	-0.79609	0.435548
16S	OTU32	L*T	Butner	UPRS	Low T	Low T	-0.46029	-1.16383	0.243255
16S	OTU32	L*T	UPRS	UPRS	High T	Low T	-0.28002	-0.82432	0.264292
16S	OTU33	L	Butner	UPRS			-0.46158	-0.85098	-0.07218
16S	OTU33	T			High T	Low T	0.075955	-0.25296	0.404871
16S	OTU33	L*T	Butner	Butner	High T	Low T	0.443623	-0.10068	0.987929
16S	OTU33	L*T	Butner	UPRS	High T	High T	-0.09391	-0.5778	0.38998
16S	OTU33	L*T	Butner	UPRS	High T	Low T	-0.38562	-0.87207	0.100821
16S	OTU33	L*T	Butner	UPRS	Low T	High T	-0.53753	-1.06951	-0.00556
16S	OTU33	L*T	Butner	UPRS	Low T	Low T	-0.82925	-1.45446	-0.20404
16S	OTU33	L*T	UPRS	UPRS	High T	Low T	-0.29171	-0.78353	0.200101
16S	OTU34	L	Butner	UPRS			-0.26659	-0.69393	0.160754
16S	OTU34	T			High T	Low T	0.098709	-0.2559	0.453321
16S	OTU34	L*T	Butner	Butner	High T	Low T	-0.2538	-0.8428	0.33519
16S	OTU34	L*T	Butner	UPRS	High T	High T	-0.6191	-1.13068	-0.10752
16S	OTU34	L*T	Butner	UPRS	High T	Low T	-0.16788	-0.69153	0.355775
16S	OTU34	L*T	Butner	UPRS	Low T	High T	-0.3653	-0.95055	0.219963
16S	OTU34	L*T	Butner	UPRS	Low T	Low T	0.085926	-0.60104	0.772897
16S	OTU34	L*T	UPRS	UPRS	High T	Low T	0.451222	-0.07126	0.9737
16S	OTU35	L	Butner	UPRS			-0.07018	-0.42131	0.280949
16S	OTU35	T			High T	Low T	-0.00724	-0.30227	0.287786
16S	OTU35	L*T	Butner	Butner	High T	Low T	0.028976	-0.45768	0.515634
16S	OTU35	L*T	Butner	UPRS	High T	High T	-0.03396	-0.45914	0.391224
16S	OTU35	L*T	Butner	UPRS	High T	Low T	-0.07742	-0.50766	0.352812
16S	OTU35	L*T	Butner	UPRS	Low T	High T	-0.06293	-0.54828	0.422415
16S	OTU35	L*T	Butner	UPRS	Low T	Low T	-0.1064	-0.67272	0.459927
16S	OTU35	L*T	UPRS	UPRS	High T	Low T	-0.04346	-0.48184	0.39491
16S	OTU36	L	Butner	UPRS			4.754671	3.128714	6.380629
16S	OTU36	T			High T	Low T	0.33761	-1.12145	1.79667

16S	OTU36	L*T	Butner	Butner	High T	Low T	-0.81066	-3.49967	1.878346
16S	OTU36	L*T	Butner	UPRS	High T	High T	3.6064	1.38034	5.832461
16S	OTU36	L*T	Butner	UPRS	High T	Low T	5.092282	3.037656	7.146907
16S	OTU36	L*T	Butner	UPRS	Low T	High T	4.417061	2.109747	6.724375
16S	OTU36	L*T	Butner	UPRS	Low T	Low T	5.902942	3.069585	8.736299
16S	OTU36	L*T	UPRS	UPRS	High T	Low T	1.485881	-0.6873	3.659067
16S	OTU37	L	Butner	UPRS			0.102183	-0.27002	0.474388
16S	OTU37	T			High T	Low T	-0.05373	-0.37353	0.266079
16S	OTU37	L*T	Butner	Butner	High T	Low T	-0.30054	-0.81227	0.211192
16S	OTU37	L*T	Butner	UPRS	High T	High T	-0.14463	-0.60723	0.317973
16S	OTU37	L*T	Butner	UPRS	High T	Low T	0.048456	-0.41727	0.514187
16S	OTU37	L*T	Butner	UPRS	Low T	High T	0.15591	-0.3586	0.670419
16S	OTU37	L*T	Butner	UPRS	Low T	Low T	0.348994	-0.23258	0.930571
16S	OTU37	L*T	UPRS	UPRS	High T	Low T	0.193084	-0.27365	0.659819
16S	OTU38	L	Butner	UPRS			0.122596	-0.68778	0.932973
16S	OTU38	T			High T	Low T	0.852965	0.14892	1.55701
16S	OTU38	L*T	Butner	Butner	High T	Low T	2.050018	0.892303	3.207733
16S	OTU38	L*T	Butner	UPRS	High T	High T	1.319649	0.362024	2.277274
16S	OTU38	L*T	Butner	UPRS	High T	Low T	0.975561	-0.00336	1.954485
16S	OTU38	L*T	Butner	UPRS	Low T	High T	-0.73037	-1.89075	0.430013
16S	OTU38	L*T	Butner	UPRS	Low T	Low T	-1.07446	-2.43185	0.282935
16S	OTU38	L*T	UPRS	UPRS	High T	Low T	-0.34409	-1.39156	0.70338
16S	OTU39	L	Butner	UPRS			0.221751	-0.48897	0.932469
16S	OTU39	T			High T	Low T	0.315438	-0.27997	0.910845
16S	OTU39	L*T	Butner	Butner	High T	Low T	0.809539	-0.17231	1.791384
16S	OTU39	L*T	Butner	UPRS	High T	High T	0.715852	-0.15155	1.583256
16S	OTU39	L*T	Butner	UPRS	High T	Low T	0.537189	-0.34708	1.421458
16S	OTU39	L*T	Butner	UPRS	Low T	High T	-0.09369	-1.06184	0.87447
16S	OTU39	L*T	Butner	UPRS	Low T	Low T	-0.27235	-1.40846	0.863764
16S	OTU39	L*T	UPRS	UPRS	High T	Low T	-0.17866	-1.06065	0.703325
16S	OTU40	L	Butner	UPRS			-0.23812	-0.80838	0.33214
16S	OTU40	T			High T	Low T	-0.27788	-0.75774	0.201987
16S	OTU40	L*T	Butner	Butner	High T	Low T	-0.36635	-1.16477	0.432061
16S	OTU40	L*T	Butner	UPRS	High T	High T	-0.3266	-1.04598	0.392793
16S	OTU40	L*T	Butner	UPRS	High T	Low T	-0.516	-1.23134	0.199336
16S	OTU40	L*T	Butner	UPRS	Low T	High T	0.039757	-0.73434	0.813859
16S	OTU40	L*T	Butner	UPRS	Low T	Low T	-0.14965	-1.07709	0.777794
16S	OTU40	L*T	UPRS	UPRS	High T	Low T	-0.18941	-0.93126	0.552451
16S	OTU41	L	Butner	UPRS			-0.12949	-0.47685	0.217872
16S	OTU41	T			High T	Low T	-0.21854	-0.51294	0.075846
16S	OTU41	L*T	Butner	Butner	High T	Low T	0.005673	-0.47447	0.485818

16S	OTU41	L*T	Butner	UPRS	High T	High T	0.09473	-0.32313	0.512592
16S	OTU41	L*T	Butner	UPRS	High T	Low T	-0.34803	-0.77132	0.075255
16S	OTU41	L*T	Butner	UPRS	Low T	High T	0.089057	-0.3962	0.574317
16S	OTU41	L*T	Butner	UPRS	Low T	Low T	-0.35371	-0.90726	0.199853
16S	OTU41	L*T	UPRS	UPRS	High T	Low T	-0.44276	-0.86998	-0.01555
16S	OTU42	L	Butner	UPRS			-0.01477	-0.53537	0.505824
16S	OTU42	T			High T	Low T	-0.068	-0.50403	0.368028
16S	OTU42	L*T	Butner	Butner	High T	Low T	0.377817	-0.33209	1.087725
16S	OTU42	L*T	Butner	UPRS	High T	High T	0.431048	-0.17155	1.033649
16S	OTU42	L*T	Butner	UPRS	High T	Low T	-0.08277	-0.70667	0.541119
16S	OTU42	L*T	Butner	UPRS	Low T	High T	0.053231	-0.67687	0.783327
16S	OTU42	L*T	Butner	UPRS	Low T	Low T	-0.46059	-1.30026	0.379075
16S	OTU42	L*T	UPRS	UPRS	High T	Low T	-0.51382	-1.14818	0.120532
16S	OTU43	L	Butner	UPRS			-1.0559	-1.54901	-0.5628
16S	OTU43	T			High T	Low T	0.241024	-0.17604	0.658088
16S	OTU43	L*T	Butner	Butner	High T	Low T	0.672524	-0.00683	1.351882
16S	OTU43	L*T	Butner	UPRS	High T	High T	-0.6244	-1.23996	-0.00885
16S	OTU43	L*T	Butner	UPRS	High T	Low T	-0.81488	-1.4322	-0.19755
16S	OTU43	L*T	Butner	UPRS	Low T	High T	-1.29693	-1.97005	-0.6238
16S	OTU43	L*T	Butner	UPRS	Low T	Low T	-1.4874	-2.27332	-0.70149
16S	OTU43	L*T	UPRS	UPRS	High T	Low T	-0.19048	-0.82025	0.439303
16S	OTU44	L	Butner	UPRS			0.224226	-0.22064	0.669089
16S	OTU44	T			High T	Low T	0.118095	-0.25043	0.486618
16S	OTU44	L*T	Butner	Butner	High T	Low T	0.420422	-0.19212	1.03296
16S	OTU44	L*T	Butner	UPRS	High T	High T	0.526552	0.002373	1.050732
16S	OTU44	L*T	Butner	UPRS	High T	Low T	0.342321	-0.19819	0.882834
16S	OTU44	L*T	Butner	UPRS	Low T	High T	0.106131	-0.50646	0.718725
16S	OTU44	L*T	Butner	UPRS	Low T	Low T	-0.0781	-0.80835	0.652145
16S	OTU44	L*T	UPRS	UPRS	High T	Low T	-0.18423	-0.73978	0.371316
16S	OTU45	L	Butner	UPRS			-1.25485	-2.05785	-0.45186
16S	OTU45	T			High T	Low T	0.186839	-0.48396	0.857641
16S	OTU45	L*T	Butner	Butner	High T	Low T	1.394643	0.313573	2.475714
16S	OTU45	L*T	Butner	UPRS	High T	High T	-0.04705	-0.96338	0.869282
16S	OTU45	L*T	Butner	UPRS	High T	Low T	-1.06801	-2.03665	-0.09938
16S	OTU45	L*T	Butner	UPRS	Low T	High T	-1.44169	-2.56031	-0.32308
16S	OTU45	L*T	Butner	UPRS	Low T	Low T	-2.46266	-3.76001	-1.1653
16S	OTU45	L*T	UPRS	UPRS	High T	Low T	-1.02097	-2.00301	-0.03892
16S	OTU46	L	Butner	UPRS			-0.0598	-0.955	0.835407
16S	OTU46	T			High T	Low T	-0.40845	-1.18701	0.370114
16S	OTU46	L*T	Butner	Butner	High T	Low T	-0.21946	-1.54488	1.105969
16S	OTU46	L*T	Butner	UPRS	High T	High T	0.129195	-1.01222	1.270612

16S	OTU46	L*T	Butner	UPRS	High T	Low T	-0.46824	-1.56865	0.632165
16S	OTU46	L*T	Butner	UPRS	Low T	High T	0.348651	-0.91792	1.615218
16S	OTU46	L*T	Butner	UPRS	Low T	Low T	-0.24879	-1.73764	1.240071
16S	OTU46	L*T	UPRS	UPRS	High T	Low T	-0.59744	-1.76889	0.574021
16S	OTU47	L	Butner	UPRS			-0.57753	-1.04169	-0.11336
16S	OTU47	T			High T	Low T	-0.10235	-0.48798	0.283273
16S	OTU47	L*T	Butner	Butner	High T	Low T	-0.08119	-0.71739	0.555009
16S	OTU47	L*T	Butner	UPRS	High T	High T	-0.55636	-1.11056	-0.00217
16S	OTU47	L*T	Butner	UPRS	High T	Low T	-0.67988	-1.24977	-0.10999
16S	OTU47	L*T	Butner	UPRS	Low T	High T	-0.47517	-1.11042	0.160071
16S	OTU47	L*T	Butner	UPRS	Low T	Low T	-0.59869	-1.35667	0.159285
16S	OTU47	L*T	UPRS	UPRS	High T	Low T	-0.12352	-0.70955	0.462514
16S	OTU48	L	Butner	UPRS			-0.67848	-1.22562	-0.13134
16S	OTU48	T			High T	Low T	-0.18753	-0.64511	0.270056
16S	OTU48	L*T	Butner	Butner	High T	Low T	-0.13133	-0.8867	0.624045
16S	OTU48	L*T	Butner	UPRS	High T	High T	-0.62228	-1.32144	0.076872
16S	OTU48	L*T	Butner	UPRS	High T	Low T	-0.86601	-1.56579	-0.16623
16S	OTU48	L*T	Butner	UPRS	Low T	High T	-0.49096	-1.21746	0.23554
16S	OTU48	L*T	Butner	UPRS	Low T	Low T	-0.73468	-1.60043	0.131067
16S	OTU48	L*T	UPRS	UPRS	High T	Low T	-0.24372	-0.94214	0.454694
16S	OTU49	L	Butner	UPRS			0.284516	-0.28964	0.858668
16S	OTU49	T			High T	Low T	-0.02318	-0.52838	0.482018
16S	OTU49	L*T	Butner	Butner	High T	Low T	-0.16168	-0.98423	0.660882
16S	OTU49	L*T	Butner	UPRS	High T	High T	0.146023	-0.55938	0.851423
16S	OTU49	L*T	Butner	UPRS	High T	Low T	0.261333	-0.45502	0.97769
16S	OTU49	L*T	Butner	UPRS	Low T	High T	0.307698	-0.5026	1.118
16S	OTU49	L*T	Butner	UPRS	Low T	Low T	0.423008	-0.47965	1.325664
16S	OTU49	L*T	UPRS	UPRS	High T	Low T	0.115309	-0.58249	0.813113
16S	OTU50	L	Butner	UPRS			-0.7513	-1.19069	-0.31191
16S	OTU50	T			High T	Low T	-0.05685	-0.42867	0.314972
16S	OTU50	L*T	Butner	Butner	High T	Low T	0.291026	-0.30972	0.891776
16S	OTU50	L*T	Butner	UPRS	High T	High T	-0.40342	-0.94136	0.134512
16S	OTU50	L*T	Butner	UPRS	High T	Low T	-0.80815	-1.35484	-0.26146
16S	OTU50	L*T	Butner	UPRS	Low T	High T	-0.69445	-1.29757	-0.09133
16S	OTU50	L*T	Butner	UPRS	Low T	Low T	-1.09917	-1.78938	-0.40896
16S	OTU50	L*T	UPRS	UPRS	High T	Low T	-0.40472	-0.94809	0.138642
ITS	OTU01	L	Butner	UPRS			3.190268	2.21687	4.163666
ITS	OTU01	T			High T	Low T	1.614199	0.70683	2.521568
ITS	OTU01	L*T	Butner	Butner	High T	Low T	2.294758	0.794255	3.795261
ITS	OTU01	L*T	Butner	UPRS	High T	High T	3.870827	2.515106	5.226547
ITS	OTU01	L*T	Butner	UPRS	High T	Low T	4.804467	3.487353	6.121581

ITS	OTU01	L*T	Butner	UPRS	Low T	High T	1.576069	0.231876	2.920262
ITS	OTU01	L*T	Butner	UPRS	Low T	Low T	2.509709	1.025067	3.994351
ITS	OTU01	L*T	UPRS	UPRS	High T	Low T	0.93364	-0.30824	2.175519
ITS	OTU02	L	Butner	UPRS			0.721124	-1.742	3.184249
ITS	OTU02	T			High T	Low T	-8.32526	-10.336	-6.31454
ITS	OTU02	L*T	Butner	Butner	High T	Low T	-9.76246	-12.6781	-6.8468
ITS	OTU02	L*T	Butner	UPRS	High T	High T	-0.71608	-3.86856	2.436411
ITS	OTU02	L*T	Butner	UPRS	High T	Low T	-7.60414	-10.9713	-4.23696
ITS	OTU02	L*T	Butner	UPRS	Low T	High T	9.046387	6.06611	12.02666
ITS	OTU02	L*T	Butner	UPRS	Low T	Low T	2.158324	-1.27239	5.589036
ITS	OTU02	L*T	UPRS	UPRS	High T	Low T	-6.88806	-9.91443	-3.8617
ITS	OTU03	L	Butner	UPRS			-3.33972	-5.88155	-0.7979
ITS	OTU03	T			High T	Low T	-4.99372	-7.11481	-2.87263
ITS	OTU03	L*T	Butner	Butner	High T	Low T	-2.51384	-5.97635	0.948671
ITS	OTU03	L*T	Butner	UPRS	High T	High T	-0.85985	-4.11629	2.396602
ITS	OTU03	L*T	Butner	UPRS	High T	Low T	-8.33344	-12.2044	-4.46252
ITS	OTU03	L*T	Butner	UPRS	Low T	High T	1.653993	-0.97959	4.287574
ITS	OTU03	L*T	Butner	UPRS	Low T	Low T	-5.8196	-9.99468	-1.64453
ITS	OTU03	L*T	UPRS	UPRS	High T	Low T	-7.4736	-10.9554	-3.99178
ITS	OTU04	L	Butner	UPRS			-1.75061	-3.12578	-0.37544
ITS	OTU04	T			High T	Low T	6.245067	4.926054	7.564079
ITS	OTU04	L*T	Butner	Butner	High T	Low T	2.249703	-0.02545	4.524854
ITS	OTU04	L*T	Butner	UPRS	High T	High T	-5.74598	-7.65838	-3.83358
ITS	OTU04	L*T	Butner	UPRS	High T	Low T	4.494453	2.536418	6.452489
ITS	OTU04	L*T	Butner	UPRS	Low T	High T	-7.99568	-9.84713	-6.14423
ITS	OTU04	L*T	Butner	UPRS	Low T	Low T	2.244751	-0.10602	4.595518
ITS	OTU04	L*T	UPRS	UPRS	High T	Low T	10.24043	8.315728	12.16513
ITS	OTU05	L	Butner	UPRS			6.239988	5.013925	7.466052
ITS	OTU05	T			High T	Low T	3.960179	2.862428	5.05793
ITS	OTU05	L*T	Butner	Butner	High T	Low T	2.048806	0.354474	3.743139
ITS	OTU05	L*T	Butner	UPRS	High T	High T	4.328615	2.939434	5.717796
ITS	OTU05	L*T	Butner	UPRS	High T	Low T	10.20017	8.372299	12.02804
ITS	OTU05	L*T	Butner	UPRS	Low T	High T	2.279809	0.839158	3.72046
ITS	OTU05	L*T	Butner	UPRS	Low T	Low T	8.151361	5.960885	10.34184
ITS	OTU05	L*T	UPRS	UPRS	High T	Low T	5.871552	4.065756	7.677348
ITS	OTU06	L	Butner	UPRS			1.685441	0.530784	2.840098
ITS	OTU06	T			High T	Low T	6.252885	5.188097	7.317673
ITS	OTU06	L*T	Butner	Butner	High T	Low T	2.463677	0.844516	4.082837
ITS	OTU06	L*T	Butner	UPRS	High T	High T	-2.10377	-3.42281	-0.78472
ITS	OTU06	L*T	Butner	UPRS	High T	Low T	7.938326	6.197595	9.679058
ITS	OTU06	L*T	Butner	UPRS	Low T	High T	-4.56744	-5.94725	-3.18764

ITS	OTU06	L*T	Butner	UPRS	Low T	Low T	5.47465	3.385467	7.563833
ITS	OTU06	L*T	UPRS	UPRS	High T	Low T	10.04209	8.28597	11.79822
ITS	OTU07	L	Butner	UPRS			-8.39264	-10.9998	-5.78543
ITS	OTU07	T			High T	Low T	-1.28569	-3.46778	0.896395
ITS	OTU07	L*T	Butner	Butner	High T	Low T	-0.35017	-4.02308	3.322753
ITS	OTU07	L*T	Butner	UPRS	High T	High T	-7.45712	-10.4832	-4.43104
ITS	OTU07	L*T	Butner	UPRS	High T	Low T	-9.67833	-12.7766	-6.58006
ITS	OTU07	L*T	Butner	UPRS	Low T	High T	-7.10695	-10.7837	-3.43016
ITS	OTU07	L*T	Butner	UPRS	Low T	Low T	-9.32816	-13.4499	-5.2064
ITS	OTU07	L*T	UPRS	UPRS	High T	Low T	-2.22122	-5.15101	0.708577
ITS	OTU08	L	Butner	UPRS			-4.57146	-7.53106	-1.61187
ITS	OTU08	T			High T	Low T	1.388788	-1.48384	4.261414
ITS	OTU08	L*T	Butner	Butner	High T	Low T	-5.1964	-9.72607	-0.66674
ITS	OTU08	L*T	Butner	UPRS	High T	High T	-11.1567	-14.8163	-7.49704
ITS	OTU08	L*T	Butner	UPRS	High T	Low T	-3.18267	-7.00475	0.639396
ITS	OTU08	L*T	Butner	UPRS	Low T	High T	-5.96025	-10.3664	-1.5541
ITS	OTU08	L*T	Butner	UPRS	Low T	Low T	2.013729	-2.0123	6.039756
ITS	OTU08	L*T	UPRS	UPRS	High T	Low T	7.97398	5.133324	10.81464
ITS	OTU10	L	Butner	UPRS			1.835527	-0.14361	3.814665
ITS	OTU10	T			High T	Low T	-2.74004	-4.38055	-1.09952
ITS	OTU10	L*T	Butner	Butner	High T	Low T	-5.13398	-8.03758	-2.23038
ITS	OTU10	L*T	Butner	UPRS	High T	High T	-0.55841	-2.93533	1.818501
ITS	OTU10	L*T	Butner	UPRS	High T	Low T	-0.90451	-3.39765	1.588625
ITS	OTU10	L*T	Butner	UPRS	Low T	High T	4.575565	1.929657	7.221474
ITS	OTU10	L*T	Butner	UPRS	Low T	Low T	4.229469	1.024188	7.43475
ITS	OTU10	L*T	UPRS	UPRS	High T	Low T	-0.3461	-2.59138	1.899183
ITS	OTU11	L	Butner	UPRS			-2.18017	-6.55924	2.198904
ITS	OTU11	T			High T	Low T	0.489002	-1.68644	2.664445
ITS	OTU11	L*T	Butner	Butner	High T	Low T	2.944639	-1.63294	7.522221
ITS	OTU11	L*T	Butner	UPRS	High T	High T	0.275467	-2.91734	3.468272
ITS	OTU11	L*T	Butner	UPRS	High T	Low T	-1.69117	-6.77698	3.394641
ITS	OTU11	L*T	Butner	UPRS	Low T	High T	-2.66917	-7.35449	2.016148
ITS	OTU11	L*T	Butner	UPRS	Low T	Low T	-4.63581	-12.6185	3.346893
ITS	OTU11	L*T	UPRS	UPRS	High T	Low T	-1.96663	-6.87334	2.940072
ITS	OTU12	L	Butner	UPRS			4.927888	3.761621	6.094156
ITS	OTU12	T			High T	Low T	4.013558	2.953139	5.073977
ITS	OTU12	L*T	Butner	Butner	High T	Low T	1.619959	-0.14078	3.380698
ITS	OTU12	L*T	Butner	UPRS	High T	High T	2.53429	1.031304	4.037275
ITS	OTU12	L*T	Butner	UPRS	High T	Low T	8.941447	7.213818	10.66907
ITS	OTU12	L*T	Butner	UPRS	Low T	High T	0.91433	-0.49444	2.323102
ITS	OTU12	L*T	Butner	UPRS	Low T	Low T	7.321487	5.195991	9.446983

ITS	OTU12	L*T	UPRS	UPRS	High T	Low T	6.407157	4.616873	8.19744
ITS	OTU13	L	Butner	UPRS			0.064204	-2.69337	2.821782
ITS	OTU13	T			High T	Low T	-2.29367	-4.13829	-0.44904
ITS	OTU13	L*T	Butner	Butner	High T	Low T	-4.59215	-8.18131	-1.00298
ITS	OTU13	L*T	Butner	UPRS	High T	High T	-2.23428	-5.13946	0.670903
ITS	OTU13	L*T	Butner	UPRS	High T	Low T	-2.22946	-5.51933	1.060404
ITS	OTU13	L*T	Butner	UPRS	Low T	High T	2.357869	-0.98736	5.703094
ITS	OTU13	L*T	Butner	UPRS	Low T	Low T	2.362684	-2.41749	7.14286
ITS	OTU13	L*T	UPRS	UPRS	High T	Low T	0.004815	-3.15823	3.167861
ITS	OTU14	L	Butner	UPRS			2.753611	0.67508	4.832143
ITS	OTU14	T			High T	Low T	3.140794	1.174715	5.106872
ITS	OTU14	L*T	Butner	Butner	High T	Low T	2.519926	-0.67407	5.713918
ITS	OTU14	L*T	Butner	UPRS	High T	High T	2.132743	-0.60988	4.875366
ITS	OTU14	L*T	Butner	UPRS	High T	Low T	5.894405	3.130734	8.658077
ITS	OTU14	L*T	Butner	UPRS	Low T	High T	-0.38718	-3.34246	2.568091
ITS	OTU14	L*T	Butner	UPRS	Low T	Low T	3.37448	-0.21847	6.96743
ITS	OTU14	L*T	UPRS	UPRS	High T	Low T	3.761662	0.70879	6.814534
ITS	OTU15	L	Butner	UPRS			-1.43835	-4.25707	1.380381
ITS	OTU15	T			High T	Low T	-2.27875	-4.9116	0.354097
ITS	OTU15	L*T	Butner	Butner	High T	Low T	4.204429	-0.09988	8.508733
ITS	OTU15	L*T	Butner	UPRS	High T	High T	5.044834	1.196281	8.893388
ITS	OTU15	L*T	Butner	UPRS	High T	Low T	-3.7171	-7.1617	-0.27249
ITS	OTU15	L*T	Butner	UPRS	Low T	High T	0.840405	-3.38912	5.069935
ITS	OTU15	L*T	Butner	UPRS	Low T	Low T	-7.92152	-12.081	-3.76207
ITS	OTU15	L*T	UPRS	UPRS	High T	Low T	-8.76193	-12.1617	-5.36212
ITS	OTU16	L	Butner	UPRS			-0.01016	-1.75645	1.736141
ITS	OTU16	T			High T	Low T	-2.15953	-3.56064	-0.75842
ITS	OTU16	L*T	Butner	Butner	High T	Low T	-5.57835	-7.83498	-3.32171
ITS	OTU16	L*T	Butner	UPRS	High T	High T	-3.42897	-5.77198	-1.08596
ITS	OTU16	L*T	Butner	UPRS	High T	Low T	-2.16969	-4.51506	0.175687
ITS	OTU16	L*T	Butner	UPRS	Low T	High T	2.149375	0.022277	4.276474
ITS	OTU16	L*T	Butner	UPRS	Low T	Low T	3.408661	1.025288	5.792033
ITS	OTU16	L*T	UPRS	UPRS	High T	Low T	1.259285	-0.71679	3.235356
ITS	OTU17	L	Butner	UPRS			-4.41321	-6.76183	-2.0646
ITS	OTU17	T			High T	Low T	-4.2323	-6.00139	-2.46322
ITS	OTU17	L*T	Butner	Butner	High T	Low T	-1.31257	-4.46854	1.843409
ITS	OTU17	L*T	Butner	UPRS	High T	High T	-1.49348	-4.37045	1.383493
ITS	OTU17	L*T	Butner	UPRS	High T	Low T	-8.64552	-11.5737	-5.71733
ITS	OTU17	L*T	Butner	UPRS	Low T	High T	-0.18091	-3.13337	2.771549
ITS	OTU17	L*T	Butner	UPRS	Low T	Low T	-7.33295	-11.2567	-3.40925
ITS	OTU17	L*T	UPRS	UPRS	High T	Low T	-7.15204	-10.142	-4.16212

ITS	OTU19	L	Butner	UPRS			-0.49093	-2.92441	1.942545
ITS	OTU19	T			High T	Low T	0.488771	-1.60468	2.58222
ITS	OTU19	L*T	Butner	Butner	High T	Low T	0.48697	-3.39444	4.368375
ITS	OTU19	L*T	Butner	UPRS	High T	High T	-0.49273	-3.43174	2.446269
ITS	OTU19	L*T	Butner	UPRS	High T	Low T	-0.00216	-2.85536	2.851037
ITS	OTU19	L*T	Butner	UPRS	Low T	High T	-0.9797	-4.5107	2.551293
ITS	OTU19	L*T	Butner	UPRS	Low T	Low T	-0.48913	-4.82111	3.842845
ITS	OTU19	L*T	UPRS	UPRS	High T	Low T	0.490572	-2.55243	3.533578
ITS	OTU20	L	Butner	UPRS			-0.11518	-4.36276	4.132398
ITS	OTU20	T			High T	Low T	7.656436	3.371684	11.94119
ITS	OTU20	L*T	Butner	Butner	High T	Low T	9.422291	-0.44257	19.28715
ITS	OTU20	L*T	Butner	UPRS	High T	High T	1.650676	-6.23061	9.531957
ITS	OTU20	L*T	Butner	UPRS	High T	Low T	7.541256	2.440694	12.64182
ITS	OTU20	L*T	Butner	UPRS	Low T	High T	-7.77161	-14.6117	-0.93156
ITS	OTU20	L*T	Butner	UPRS	Low T	Low T	-1.88103	-11.0961	7.334004
ITS	OTU20	L*T	UPRS	UPRS	High T	Low T	5.890581	-1.20522	12.98638
ITS	OTU21	L	Butner	UPRS			3.356127	-1.07985	7.792104
ITS	OTU21	T			High T	Low T	-4.19734	-10.337	1.942278
ITS	OTU21	L*T	Butner	Butner	High T	Low T	-9.51924	-21.8529	2.814426
ITS	OTU21	L*T	Butner	UPRS	High T	High T	-1.96577	-8.38614	4.454587
ITS	OTU21	L*T	Butner	UPRS	High T	Low T	-0.84122	-6.60062	4.918188
ITS	OTU21	L*T	Butner	UPRS	Low T	High T	7.553469	-1.47842	16.58535
ITS	OTU21	L*T	Butner	UPRS	Low T	Low T	8.678028	-1.28618	18.64223
ITS	OTU21	L*T	UPRS	UPRS	High T	Low T	1.124559	-3.81721	6.066326
ITS	OTU22	L	Butner	UPRS			1.914468	-0.27924	4.108176
ITS	OTU22	T			High T	Low T	2.39206	0.472757	4.311363
ITS	OTU22	L*T	Butner	Butner	High T	Low T	2.000524	-0.86581	4.866859
ITS	OTU22	L*T	Butner	UPRS	High T	High T	1.522932	-1.14778	4.193641
ITS	OTU22	L*T	Butner	UPRS	High T	Low T	4.306529	1.227161	7.385896
ITS	OTU22	L*T	Butner	UPRS	Low T	High T	-0.47759	-3.21797	2.262782
ITS	OTU22	L*T	Butner	UPRS	Low T	Low T	2.306005	-1.51122	6.123228
ITS	OTU22	L*T	UPRS	UPRS	High T	Low T	2.783597	-0.56763	6.134827
ITS	OTU24	L	Butner	UPRS			2.428219	-0.42562	5.282057
ITS	OTU24	T			High T	Low T	-1.94012	-3.57703	-0.30321
ITS	OTU24	L*T	Butner	Butner	High T	Low T	-3.16584	-6.5054	0.173711
ITS	OTU24	L*T	Butner	UPRS	High T	High T	1.2025	-1.43301	3.838007
ITS	OTU24	L*T	Butner	UPRS	High T	Low T	0.488095	-2.89978	3.875966
ITS	OTU24	L*T	Butner	UPRS	Low T	High T	4.368342	1.179286	7.557398
ITS	OTU24	L*T	Butner	UPRS	Low T	Low T	3.653937	-1.278	8.585877
ITS	OTU24	L*T	UPRS	UPRS	High T	Low T	-0.71441	-3.74549	2.31668
ITS	OTU25	L	Butner	UPRS			2.773539	1.679278	3.867799

ITS	OTU25	T			High T	Low T	0.907391	-0.08462	1.899402
ITS	OTU25	L*T	Butner	Butner	High T	Low T	0.946913	-0.7276	2.621425
ITS	OTU25	L*T	Butner	UPRS	High T	High T	2.813061	1.305373	4.320749
ITS	OTU25	L*T	Butner	UPRS	High T	Low T	3.680929	2.205634	5.156225
ITS	OTU25	L*T	Butner	UPRS	Low T	High T	1.866148	0.387471	3.344825
ITS	OTU25	L*T	Butner	UPRS	Low T	Low T	2.734016	0.968074	4.499958
ITS	OTU25	L*T	UPRS	UPRS	High T	Low T	0.867868	-0.60218	2.337918
ITS	OTU26	L	Butner	UPRS			2.422079	1.19623	3.647928
ITS	OTU26	T			High T	Low T	1.660009	0.563667	2.756351
ITS	OTU26	L*T	Butner	Butner	High T	Low T	1.521485	-0.17799	3.220958
ITS	OTU26	L*T	Butner	UPRS	High T	High T	2.283555	0.561402	4.005709
ITS	OTU26	L*T	Butner	UPRS	High T	Low T	4.082088	2.391451	5.772725
ITS	OTU26	L*T	Butner	UPRS	Low T	High T	0.76207	-0.83514	2.359284
ITS	OTU26	L*T	Butner	UPRS	Low T	Low T	2.560603	0.688232	4.432974
ITS	OTU26	L*T	UPRS	UPRS	High T	Low T	1.798533	0.071715	3.525351
ITS	OTU27	L	Butner	UPRS			-1.63414	-3.16342	-0.10487
ITS	OTU27	T			High T	Low T	2.643318	1.316042	3.970594
ITS	OTU27	L*T	Butner	Butner	High T	Low T	0.534329	-1.68549	2.754153
ITS	OTU27	L*T	Butner	UPRS	High T	High T	-3.74313	-5.64751	-1.83875
ITS	OTU27	L*T	Butner	UPRS	High T	Low T	1.009175	-0.98701	3.005356
ITS	OTU27	L*T	Butner	UPRS	Low T	High T	-4.27746	-6.33074	-2.22418
ITS	OTU27	L*T	Butner	UPRS	Low T	Low T	0.474846	-2.07752	3.027216
ITS	OTU27	L*T	UPRS	UPRS	High T	Low T	4.752307	2.737463	6.76715
ITS	OTU31	L	Butner	UPRS			0.358207	-1.00641	1.722828
ITS	OTU31	T			High T	Low T	1.573575	0.37325	2.7739
ITS	OTU31	L*T	Butner	Butner	High T	Low T	2.013661	0.16418	3.863142
ITS	OTU31	L*T	Butner	UPRS	High T	High T	0.798293	-0.85141	2.447995
ITS	OTU31	L*T	Butner	UPRS	High T	Low T	1.931781	0.175402	3.68816
ITS	OTU31	L*T	Butner	UPRS	Low T	High T	-1.21537	-3.09182	0.661085
ITS	OTU31	L*T	Butner	UPRS	Low T	Low T	-0.08188	-2.16037	1.996615
ITS	OTU31	L*T	UPRS	UPRS	High T	Low T	1.133489	-0.53332	2.800298
ITS	OTU34	L	Butner	UPRS			4.744189	2.457091	7.031286
ITS	OTU34	T			High T	Low T	-0.04225	-1.80247	1.717974
ITS	OTU34	L*T	Butner	Butner	High T	Low T	0.965479	-1.72313	3.654087
ITS	OTU34	L*T	Butner	UPRS	High T	High T	5.751913	3.184043	8.319784
ITS	OTU34	L*T	Butner	UPRS	High T	Low T	4.701943	1.654345	7.749541
ITS	OTU34	L*T	Butner	UPRS	Low T	High T	4.786434	2.071563	7.501305
ITS	OTU34	L*T	Butner	UPRS	Low T	Low T	3.736464	-0.09492	7.56785
ITS	OTU34	L*T	UPRS	UPRS	High T	Low T	-1.04997	-4.17727	2.077326
ITS	OTU36	L	Butner	UPRS			0.042844	-8.32204	8.407731
ITS	OTU36	T			High T	Low T	3.873232	-1.29265	9.039115

ITS	OTU36	L*T	Butner	Butner	High T	Low T	4.229728	-8.17034	16.62979
ITS	OTU36	L*T	Butner	UPRS	High T	High T	0.399339	-8.78095	9.579629
ITS	OTU36	L*T	Butner	UPRS	High T	Low T	3.916076	-7.26048	15.09264
ITS	OTU36	L*T	Butner	UPRS	Low T	High T	-3.83039	-12.1008	4.440034
ITS	OTU36	L*T	Butner	UPRS	Low T	Low T	-0.31365	-17.1531	16.52585
ITS	OTU36	L*T	UPRS	UPRS	High T	Low T	3.516737	-7.77551	14.80898
ITS	OTU38	L	Butner	UPRS			2.694065	1.325001	4.06313
ITS	OTU38	T			High T	Low T	3.574748	2.228328	4.921167
ITS	OTU38	L*T	Butner	Butner	High T	Low T	1.920035	-0.24417	4.084245
ITS	OTU38	L*T	Butner	UPRS	High T	High T	1.039353	-0.82489	2.903601
ITS	OTU38	L*T	Butner	UPRS	High T	Low T	6.268813	4.22241	8.315216
ITS	OTU38	L*T	Butner	UPRS	Low T	High T	-0.88068	-2.66579	0.904423
ITS	OTU38	L*T	Butner	UPRS	Low T	Low T	4.348778	2.117696	6.579859
ITS	OTU38	L*T	UPRS	UPRS	High T	Low T	5.22946	3.319916	7.139004
ITS	OTU42	L	Butner	UPRS			0.483868	-1.72407	2.691809
ITS	OTU42	T			High T	Low T	0.269036	-1.50839	2.04646
ITS	OTU42	L*T	Butner	Butner	High T	Low T	0.925404	-2.82725	4.678057
ITS	OTU42	L*T	Butner	UPRS	High T	High T	1.140236	-1.92212	4.202591
ITS	OTU42	L*T	Butner	UPRS	High T	Low T	0.752904	-1.96311	3.468915
ITS	OTU42	L*T	Butner	UPRS	Low T	High T	0.214831	-2.73335	3.16301
ITS	OTU42	L*T	Butner	UPRS	Low T	Low T	-0.1725	-4.62848	4.283474
ITS	OTU42	L*T	UPRS	UPRS	High T	Low T	-0.38733	-3.81075	3.036086
ITS	OTU44	L	Butner	UPRS			4.075408	-0.49875	8.649562
ITS	OTU44	T			High T	Low T	4.56962	-0.28045	9.419691
ITS	OTU44	L*T	Butner	Butner	High T	Low T	7.848536	1.301644	14.39543
ITS	OTU44	L*T	Butner	UPRS	High T	High T	7.354323	1.029781	13.67886
ITS	OTU44	L*T	Butner	UPRS	High T	Low T	8.645028	2.165352	15.1247
ITS	OTU44	L*T	Butner	UPRS	Low T	High T	-0.49421	-7.343	6.354579
ITS	OTU44	L*T	Butner	UPRS	Low T	Low T	0.796492	-5.24794	6.840927
ITS	OTU44	L*T	UPRS	UPRS	High T	Low T	1.290705	-4.94418	7.525594
ITS	OTU49	L	Butner	UPRS			2.134644	0.87893	3.390358
ITS	OTU49	T			High T	Low T	3.655169	2.469145	4.841192
ITS	OTU49	L*T	Butner	Butner	High T	Low T	2.67762	0.97343	4.381811
ITS	OTU49	L*T	Butner	UPRS	High T	High T	1.157095	-0.35214	2.666326
ITS	OTU49	L*T	Butner	UPRS	High T	Low T	5.789813	3.950667	7.628958
ITS	OTU49	L*T	Butner	UPRS	Low T	High T	-1.52052	-3.12816	0.087108
ITS	OTU49	L*T	Butner	UPRS	Low T	Low T	3.112192	1.101708	5.122676
ITS	OTU49	L*T	UPRS	UPRS	High T	Low T	4.632717	2.879085	6.386349
