# Supplementary material for: Beef cattle that respond differently to fescue toxicosis have distinct gastrointestinal tract microbiota
Source: PLoS One. 2020 Jul 23;15(7):e0229192. doi: 10.1371/journal.pone.0229192 (PMC7377488; doi:10.1371/journal.pone.0229192)
Supplement: S3 Table — (PDF) [file pone.0229192.s005.pdf]

Table S3. Residual variance (σ_e^2)[1] for each window period (WP), and AWG_res[2] means for each WP by genetic group (GG)

WP	σ_e^2	GG[3]	
		HT	LT
1_7	6.0001^{A} (0.7096)	3.74^{a} (0.26)	-3.52^{c} (0.26)
1_13	0.0707^{C} (0.008)	0.29^{b} (0.26)	-0.29^{b} (0.26)
7_13	2.9418^{B} (0.348)	-0.03^{b} (0.26)	0.05^{b} (0.26)

[1]Estimated from Eq.2 and expressed as (kg/week)^2;

[2]Residuals of average weekly gain (from Eq. 1, analyzed with Eq. 3). Expressed as kg/week;

[3]HT, high tolerance; LT, low tolerance;

[A-C]Variances lacking common superscripts are statistically different (*P<0.001*) based on F-test;

[a-c]Least-squares means of AWG_res lacking common superscripts are statistically different (*P<0.001*) based of Tukey's test;

Standard errors within parentheses.
