# Supplementary material for: Beef cattle that respond differently to fescue toxicosis have distinct gastrointestinal tract microbiota
Source: PLoS One. 2020 Jul 23;15(7):e0229192. doi: 10.1371/journal.pone.0229192 (PMC7377488; doi:10.1371/journal.pone.0229192)
Supplement: S4 Table — (PDF) [file pone.0229192.s006.pdf]

Table S4: The 50 most abundant 16S rRNA gene OTUs

OTU	Number of sequences	Relative abundance (%) LT	Relative abundance (%) HT	Phylum	Taxonomy (Silva v138), NCBI best BLAST hit and GenBank accession no., similarity (%)
OTU00001	289,015	8.2	6.1	*Firmicutes*	*Oscillospiraceae* UCG-005 (99.6%), *Oscillibacter* sp. strain 128x MK287653.1, 91.3%
OTU00002	241,040	1.3	11.4	*Firmicutes*	*Solibacillus* (99.6%), *Solibacillus silvestris* strain UF03 KF717516.1, 100%
OTU00003	185,984	1.3	8.5	*Proteobacteria*	*Acinetobacter* (100%), *Acinetobacter lwoffii* strain P40 MT323129.1, 100%
OTU00004	128,365	1.6	5.1	*Firmicutes*	*Bacillus* (98%), *Bacillus* sp. DSL09-2 AB576893.1, 99.6%
OTU00005	91,422	2.6	2.0	*Firmicutes*	*Oscillospiraceae* UCG-005 (100%), *Monoglobus pectinilyticus* strain ASD1037 MK615117.1, 93.7%
OTU00006	79,040	2.7	1.4	*Firmicutes*	*Peptostreptococcaceae* (100%), *Romboutsia* sp. CE17 CP051144.1, 100%
OTU00007	74,814	1.8	1.9	*Bacteroidetes*	*Prevotellaceae* UCG-004 (88.5%), *Bacteroides gallinarum* strain Bg MT152630.1, 88.6%
OTU00008	55,633	1.5	1.3	*Bacteroidetes*	*Bacteroides* (92.1%), *Bacteroides* sp. Marseille-P3684 LT985388.1, 92.5%
OTU00009	55,207	1.5	1.2	*Firmicutes*	*Planococcaceae* (99.6%), *Lysinibacillus sphaericus* strain KCCM 35418 CP026120.1, 100%
OTU00010	45,924	1.2	1.0	*Bacteroidetes*	*Rikenellaceae* RC9 gut group (90.5%)
OTU00011	45,651	1.4	1.0	*Firmicutes*	*Peptostreptococcaceae* (100%), *Enterococcus faecalis* strain BFFF11 CP045918.1, 100%
OTU00012	36,880	1.0	0.8	*Firmicutes*	*Oscillospiraceae* UCG-005 (100%), *Oscillibacter* sp. strain 128x MK287653.1, 95.3%
OTU00013	36,595	0.9	0.9	*Bacteroidetes*	*Bacteroides* (92.5%), *Bacteroides plebeius* DSM 17135 NR_041277.1, 92.5%
OTU00014	34,271	1.0	0.6	*Firmicutes*	*Planococcaceae* (100%), *Lysinibacillus pakistanensis* strain J20BS9 MT415995.1, 100%
OTU00015	33,558	1.2	0.5	*Firmicutes*	*Peptostreptococcaceae* (99.2%), *Clostridioides difficile* strain TW11-RT078 CP035499.1, 99.2%
OTU00016	30,900	0.8	0.7	*Firmicutes*	*Oscillospiraceae* UCG-005 (98.8%), *Oscillibacter* sp. strain 128x MK287653.1, 90.1%
OTU00017	30,226	0.7	0.8	*Bacteroidetes*	*Rikenellaceae* RC9 gut group (91.7%)
OTU00018	25,949	0.9	0.4	*Firmicutes*	*Clostridia* (99.2%), *Clostridiales* bacterium P40 AB730752.1, 93.68%
OTU00019	23,250	0.7	0.5	*Bacteroidetes*	*Rikenellaceae* RC9 gut group (90.9%)
OTU00020	20,683	0.7	0.4	*Firmicutes*	*Mogibacterium* (98.8%), *Mogibacterium timidum* strain KCOM MT242492.1, 94.5%
OTU00021	20,366	0.6	0.5	*Firmicutes*	uncultured *Ruminococcaceae* (92.5%), *Ruminococcus* sp. strain FBDS KY643811.1, 91.7%
OTU00022	20,316	0.1	0.9	*Firmicutes*	*Planococcaceae* (99.6%), *Psychrobacillus psychrodurans* strain EB313 MH127767.1, 100%
OTU00023	20,114	0.5	0.5	*Bacteroidetes*	*Alistipes* (94.9%), *Alistipes finegoldii* strain DSM 17242 NR_102944.1, 96.1%
OTU00024	19,425	0.5	0.5	*Bacteroidetes*	*Rikenellaceae* RC9 gut group (90.9%),

OTU00025	19,212	0.9	0.1	*Actinobacteria*	*Corynebacterium* (99.6%), *Corynebacterium kutscheri* strain NCTC3655 LR134407.1, 99.6%
OTU00026	19,171	0.7	0.3	*Euryarchaeota*	*Methanobrevibacter* (99.2%), *Methanobrevibacter* sp. YE315 CP010834.1, 100%
OTU00027	18,306	0.3	0.6	*Bacteroidetes*	*Bacteroidales* p-2534-18B5 gut group (86.6%), *Anaerocella delicata* strain WN081 NR_132392.1, 87.8%
OTU00028	18,196	0.5	0.4	*Bacteroidetes*	*Bacteroides* (92.1%), *Bacteroides coprocola* DSM 17136 NR_041278.1, 92.1%
OTU00029	16,881	0.5	0.3	*Firmicutes*	*Oscillospiraceae* UCG-005 (97.2%), *Oscillibacter* sp. strain 128x MK287653.1, 94.5%
OTU00030	16,430	0.5	0.3	*Firmicutes*	*Christensenellaceae* R-7 group (96%), Bacterium R2 JN688050.1, 97.2%
OTU00031	16,351	0.5	0.3	*Bacteroidetes*	*Anaerocella delicata* strain WN081 NR_132392.1, 87.1%
OTU00032	16,174	0.5	0.3	*Firmicutes*	*Christensenellaceae* R-7 group (95.3%), Bacterium AC2043 KF697809.1, 95.3%
OTU00033	16,164	0.4	0.4	*Bacteroidetes*	*Rikenellaceae* RC9 gut group (91.3%)
OTU00034	15,214	0.4	0.4	*Bacteroidetes*	*Rikenellaceae* dgA-11 gut group (89.3%)
OTU00035	15,190	0.4	0.4	*Bacteroidetes*	*Bacteroides* (94.9%), *Bacteroides plebeius* DSM 17135 NR_041277.1, 94.9%
OTU00036	15,148	0.2	0.6	*Actinobacteria*	*Arthrobacter* (100%), *Arthrobacter citreus* strain Y34A9 MT367753.1, 100%
OTU00037	15,099	0.4	0.4	*Firmicutes*	*Butyricicoccaceae* UCG-009 (93.7%), *Papillibacter cinnamivorans* strain CIN1 NR_025025.1, 93.7%
OTU00038	15,024	0.5	0.2	*Firmicutes*	*Clostridia* (100%), Rumen bacterium strain XS0-10 MH594342.1, 100%
OTU00039	14,711	0.4	0.4	*Bacteroidetes*	*Prevotellaceae* UCG-003 (92%), *Alloprevotella* sp. feline oral taxon 309 KM462157.1, 93.7%
OTU00040	13,842	0.4	0.3	*Firmicutes*	uncultured *Lachnospiraceae* (99.2%), *Anaerotignum faecicola* strain KGMB03357 NR_165727.1, 98%
OTU00041	13,733	0.4	0.3	*Bacteroidetes*	*Alistipes* (94.9%), *Alistipes finegoldii* strain DSM 17242 NR_102944.1, 95.7%
OTU00042	13,493	0.4	0.2	*Firmicutes*	*Christensenellaceae* R-7 group (96.8%), Bacterium AC2043 KF697809.1, 96.1%
OTU00043	13,473	0.4	0.3	*Firmicutes*	*Christensenellaceae* R-7 group (96.8%), *Christensenellaceae* bacterium LC333712.1, 97.6%
OTU00044	13,284	0.4	0.3	*Firmicutes*	*Anaerovoracaceae* Family XIII AD3011 group (97.6%), *Emergencia timonensis* strain SN18 NR_144737.1, 95.2%
OTU00045	13,150	0.5	0.1	*Firmicutes*	*Lachnospiraceae* NK3A20 group (99.6%), Bacterium strain DSM _11001 MN336244.1, 99.6%
OTU00046	12,955	0.3	0.3	*Firmicutes*	*Planococcaceae* (99.2%), *Solibacillus isronensis* strain Ba2SD-3 MT373594.1, 99.2%
OTU00047	12,948	0.3	0.3	*Firmicutes*	*Bacteria* (93.7%), *Monoglobus pectinilyticus* strain ASD1037 MK615117.1, 92.9%
OTU00048	12,924	0.3	0.3	*Bacteroidetes*	*Rikenellaceae* RC9 gut group (92.5%), %)
OTU00049	12,471	0.3	0.4	*Bacteroidetes*	*Rikenellaceae* dgA-11 gut group (89.7%), %)
OTU00050	12,466	0.4	0.2	*Tenericutes*	*Mollicutes RF39* (98%), *Spiroplasma velocicrescens* strain MQ-4 NR_025713.1, 87.7%
