# Supplementary material for: Beef cattle that respond differently to fescue toxicosis have distinct gastrointestinal tract microbiota
Source: PLoS One. 2020 Jul 23;15(7):e0229192. doi: 10.1371/journal.pone.0229192 (PMC7377488; doi:10.1371/journal.pone.0229192)
Supplement: S5 Table — (PDF) [file pone.0229192.s007.pdf]

Table S5: The 50 most abundant ITS1 OTUs

OTU	Number of sequences	Relative abundance (%) HT	Relative abundance (%) LT	UNITE (V8) Classification	Taxonomy (NCBI Blast), GenBank accession no., similarity (%)
OTU00001	50,275	17	8.6	*Pleosporales* MH633965 (99.6%)	*Microsphaeropsis arundinis*; AB775571; 99%
OTU00002	25,250	0	13.2	*Thelebolus* KP714650 (100%)	*Thelebolus sp.* MKOTU58; KP714650; 100%
OTU00003	23,768	2.1	10.3	*Thelebolus* KP714650 (99.6%)	*Thelebolus sp.* MKOTU58; KP714650; 99%
OTU00004	15,241	7.6	0	*Ascomycota* KP698358 (100%)	*Pleosporales sp.* 418H; HE653990; 100%
OTU00005	11,844	3.6	2.4	*Letendraea* LT796897 (99.6%)	*Microsphaeropsis sp.* JSM 06261702; KY086256; 98%
OTU00006	11,448	5.5	0.2	*Psilocybe* KJ182745 (100%)	*Psilocybe cf. subviscida/crobula* X-45; KC176337; 95%
OTU00007	11,030	1.1	4.6	*Thelebolus* KP714650 (99.6%)	*Thelebolus sp*. MKOTU58; KP714650; 99%
OTU00008	11,003	5.5	0	*Thelebolus* KP714650 (99.6%)	*Thelebolus sp*. MKOTU58; KP714650; 99%
OTU00009	9,407	0.6	4.3	*Pyronemataceae* FJ553261 (99.6%)	*Pyronemataceae sp.* F79; KT809108; 98%
OTU00010	8,690	0	4.6	*Thelebolus* KP714650 (99.6%)	*Thelebolus sp*. MKOTU58; KP714650; 99%
OTU00011	7,540	1.7	2.1	*Pyrenochaetopsis leptospora* MF795793 (99.6%)	*Pyrenochaetopsis leptospora* strain CBS 101635; MF795793; 99%
OTU00012	7,220	2.4	1.2	*Letendraea* LT796908 (99.6%)	*Microsphaeropsis* sp. S4A1ACS; KY305064; 99%
OTU00013	6,955	0.1	3.5	*Thelebolus* KP714650 (99.6%)	*Thelebolus sp.* MKOTU58; KP714650; 99%
OTU00014	4,714	1.8	0.5	*Orpinomyces* MK036695 (100%)	*Orpinomyces sp.* LT-3; JF747595; 100%
OTU00015	4,666	0	2.4	*Pyrenochaetopsis leptospora* MF795793 (99.2%)	*Pyrenochaetopsis leptospora* strain CBS 101635; MF795793; 99%
OTU00016	4,298	0	2.2	*Thelebolus* KP714650 (99.3%)	*Thelebolus sp.* MKOTU58; KP714650; 99%
OTU00017	4,034	0	2.1	*Thelebolus* KP714650 (100%)	*Thelebolus sp*. MKOTU58; KP714650; 100%
OTU00018	3,593	0	1.9	*Thelebolus* KP714650 (99.3%)	*Thelebolus sp*. MKOTU58; KP714650; 99%
OTU00019	2,790	0.8	0.7	*Phaeosphaeriopsis* (99.6%)	*Phaeosphaeriopsis* TMS-2011; HQ630983; 99%
OTU00020	2,771	1.1	0.3	*Pyrenochaetopsis leptospora* MF795793 (99.6%)	*Pyrenochaetopsis leptospora* strain CBS 101635; MF795793; 99%
OTU00021	2,717	1.1	0.2	*Pyrenochaetopsis leptospora* (99.6%)	*Pyrenochaetopsis leptospora* strain CBS 101635; MF795793; 99%
OTU00022	2,679	1.1	0.3	*Orpinomyces* MK036685 (99.209%)	*Orpinomyces sp.* LT-7; JF747596; 99%
OTU00023	2,486	0.9	0.3	*Didymocyrtis cladoniicola* LT796886 (100%)	*Pleosporales sp*. MH179; FM995624; 99%
OTU00024	2,367	0.2	1	*Coprinopsis cothurnata* AY461833 (100%)	*Coprinopsis cothurnata* SFSU DEH548; AY461833; 100%

OTU00025	2,268	0.6	0.6	*Caecomyces* AB334759 (93.9%)	*Caecomyces* sp. CR4; AB334759; 94%
OTU00026	2,004	0.7	0.3	*Caecomyces* AB334759 (100%)	*Caecomyces* sp. CR4; AB334759; 100%
OTU00027	1,863	0.9	0.1	*Melanotus caricicola* (94.2%)	*Deconica sp.* PB-2016b; KX017213; 92%
OTU00028	1,669	0.4	0.5	*Pyrenochaetopsis leptospora* MF795793 (99.6%)	*Pyrenochaetopsis leptospora* strain CBS 101635; MF795793; 99%
OTU00029	1,664	0.8	0	*Pyrenochaetopsis leptospora* MF795793 (99.6%)	*Pyrenochaetopsis leptospora* strain CBS 101635; MF795793; 99%
OTU00030	1,657	0.8	0	*Preussia* KX611026 (100%)	*Preussia sp.* JJP-2009a; FJ210518; 99%
OTU00031	1,627	0.5	0.3	*Cyllamyces* GQ850314 (96%)	*Caecomyces sp.* NZB7; JF423621; 87%
OTU00032	1,622	0	0.9	*Pyrenochaetopsis leptospora* MF795793 (99.2%)	*Pleosporales sp.* NWHC; KX148619; 100%
OTU00033	1,609	0.8	0	*Pyrenochaetopsis leptospora* MF795793 (99.6%)	*Pyrenochaetopsis leptospora* strain CBS 101635; MF795793; 99%
OTU00034	1,506	0.4	0.3	*Orpinomyces* JN560954 (98.4%)	*Orpinomyces* sp. NIANP59; EU150190; 98%
OTU00035	1,492	0.3	0.5	*Neocallimastigaceae* JF274494 (91.5%)	*Orpinomyces* sp. LT-7; JF747596; 100%
OTU00036	1,467	0.6	0.1	*Phaeosphaeriopsis* KF800186 (99.6%)	*Phaeosphaeriopsis* TMS-2011; HQ630983; 99%
OTU00037	1,438	0.1	0.7	*Preussia* FJ210520 (99.6%)	*Preussia sp.* JJP-2009a; FJ210518; 99%
OTU00038	1,414	0.5	0.2	uncultured fungi FJ758183 (100%)	*Pleosporales sp.* 1 TMS-2011; HQ631002; 92%
OTU00039	1,322	0.5	0.2	*Paraphaeosphaeria michotii* JF340243 (99.3%)	*Paraphaeosphaeria michotii* M15; JF340243; 99%
OTU00040	1,317	0	0.7	*Thelebolus* KP714650 (99.6%)	*Thelebolus sp.* MKOTU58; KP714650; 99%
OTU00041	1,298	0.6	0	*Preussia* FJ210520 (100%)	*Preussia sp.* JJP-2009a; FJ210520; 100%
OTU00042	1,288	0.3	0.3	*Pyrenochaetopsis* KF800437 (96.1%)	*Pyrenochaetopsis sp.* PG293; AB916515; 96%
OTU00043	1,257	0.5	0.2	uncultured fungi JX371448 (100%)	*Podospora decipiens* strain CBS 258.69; KX171946; 99%
OTU00044	1,225	0.6	0	*Preussia* FJ210520 (100%)	*Preussia sp.* JJP-2009a; FJ210520; 100%
OTU00045	1,168	0.3	0.3	*Neocallimastigaceae* AM690084 (98%)	*Caecomyces sp.* CR4; AB334759; 90%
OTU00046	1,152	0	0.6	*Thelebolus* MK529893 (99.6%)	*Thelebolus sp.* MKOTU58; KP714650; 99%
OTU00047	1,151	0	0.6	*Chaetothyriales* HQ607986 (100%)	*Podospora cochleariformis* strain CBS 249.71; AY999123; 97%
OTU00048	1,068	0.1	0.5	*Pyronemataceae* FJ553261 (99.3%)	*Pyronemataceae sp.* F79; KT809108; 98%
OTU00049	1,016	0.4	0.1	unclassified Ascomycota JX545197 (96.2%)	*Pleosporales sp.* 5 TMS-2011; HQ631052; 90%
OTU00050	998	0.5	0	*Pleosporales* MH633965 (99.6%)	*Podospora myriaspora* strain TJ12; EU888913; 100%
