# Supplementary material for: Beef cattle that respond differently to fescue toxicosis have distinct gastrointestinal tract microbiota
Source: PLoS One. 2020 Jul 23;15(7):e0229192. doi: 10.1371/journal.pone.0229192 (PMC7377488; doi:10.1371/journal.pone.0229192)
Supplement: Attachment — Submitted filename: Response to Reviewer comments R2.pdf [file pone.0229192.s009.pdf]

Reviewer #2:
Ln63-64: Re-word it. It is a bit confusing.
Response: *As suggested, we have reworded this sentence and hope it is clearer now.*

Ln259: re-write. Confusing. It reads like there was a significant interaction of T and the main effect of T.
Response*: Changed as suggested.*

Ln260-266: This may just be a result of the ruminal adaptation of animals to the diet. It is questionable if using the w1-7 is the best option in the long term.
Response*: It could be that the underlying biological process that resulted in large variability in response to FT is indeed the ability of each animal adapting to the diet. This, therefore, could actually have a genetic basis. However, this area of research is still limited, and we hope our results can help others building more knowledge blocks on top of it so one day we can understand the reasons why there is large individual variability in response to FT. With regards to using 1_7, our decision is based on the extensive literature about variation to stressors, where it is well known that the period of larger variability indicates the most extreme stress period.*

Ln318: "However, . we"
Response*: changed as suggested.*

Results section. A T*L interaction should not make it difficult to interpret. As there are differences in the Alkaloids in total concentration and composition between sites, which could ultimately be promoting those differences. For example, for the Chao and ACE the interaction you have shows that there is no difference between HT and LT for the BBCFL location but there was a difference between HT and LT groups for the UPRS location. This interaction result description needs to be improved.
Response: *As suggested by both reviewers in the first round of revisions of this manuscript, we have changed this section to give more priority on reporting the interaction effects over the T or L effects alone. We have thus modified this section accordingly and included more general discussion regarding possible reasons for the differences in diversity and abundance in the discussion (L539-548). However, as we wanted to be cautious and try not to "overinterpret" our results, we have changed this section to accommodate the reviewer's comment, but also worded this cautiously as we cannot easily discern which effect is driving the T*L interaction effect differences observed.*

Ln482-484: where? Rumen, fecal??
Response: *In fecal samples – changed accordingly.*

L497-498: it is not difficult. You have huge differences in alkaloids concentration and composition between locations. You need to discuss how that is affecting your results.
Response*:* Thank you for the comment. Although the differences in toxin levels at each farm are largely different, the difference in abundance noted in this OTU may also be caused by different management practices or climate at the different locations, as OTU15 had opposing effects for location. Without a significant main effect or similar patterns seen in the location to explain the interaction, we feel it may be misleading to readers to explain this difference based on toxin levels alone. We thus prefer to be not too speculative about possible reasons for the differences in abundance of this OTU.

Reviewer #3:

ABSTRACT

Pag 2, ,lines 31-33: “20 HT and 20 LT cattle balanced by farm were selected fo 16S rRNA gene and ITS1 region Illumina MiSeq amplicon sequencing to compare the fecal microbiota of the two tolerance groups.”

This mind of information is quite omitted in other studies in this field of research. Here, the authors are concerned with simply and objectively describing the procedures from rRNA analysis.

Response: *We changed this sentence to more simply and objectively describe the approach in the abstract.*

Pag 2, lines 38-39: “This study also found more pronounced shifts in the microbiota in animals receiving higher amounts of the toxin.”

It is a relevant question, since the authors reported all bioethics procedures used under this kind of experiment.

Response: *Thank you for your comment.*

Pag 2, lines 41-42: “Our results thus suggest that some fungal phylotypes might be involved in mitigating fescue toxicosis.”

Maybe the aims of this manuscript would be better described in order to be compared with reported conclusion.

Response: *As suggested, we have included a sentence in the abstract of the revised manuscript highlighting the aims of the study (L28 in the revised manuscript).*

INTRODUCTION

Pag 3, lines 52-53: “However, until now, there is no clear evidence that ergovaline is the most or only responsible ergot alkaloid inducing FT – other ergot alkaloids may also contribute to FT.”

Maybe these issues would be better highlighted and associated with the tested hypothesis of the present manuscript. However, the complement of this text is high explicative and supplies the mentioned comment.

Response: *Thank you for your comment. As suggested by the reviewer above, we have clarified the aim of the study in the abstract. We hope new research can continue helping all of us better understanding all factors associated with FT.*

Pag 3, lines 61-63: “Additionally, researchers focused on the endophyte, identifying strains that produce lower levels of the ergot alkaloids while still providing drought and insect resistance for the grass”

I suggest including relevant references if the same are suitable to be exploited over here.

Response: *As suggested we have included three relevant references in the revised manuscript.*

Pag 4-5, lines 89-91: “Our goal was to identify shifts in bacterial, archaeal, and fungal microbial populations 90 (using 16S rRNA gene and ITS1 region amplicon sequencing, respectively) between the two tolerance groups across two different locations.”

The term “Our goal was” would be better exploited in the ABSTRACT section.

Response: *Similar to what the reviewer suggested before, we have now included the aim of this study in the abstract.*

MATERIALS AND METHODS

Line 129: If the residual term is presented under an algebraic way, i.e. eijk, the correct notation is the following: eijk ~ N (0, Sig2e). In fact, there is no reason to applied matrix notation over here “where I is the identity matrix. Statistical analysis was performed” (such as at line 129)

Response: *As suggested, we have reworded this sentence and removed the reference to the identity matrix.*

Pags 6-7, lines 135-137: “The aim of this study was to compare 136 the fecal microbiota of those animals that showed most extremes in their 137 performance (based on AWG), to achieve a clearer biological signal.”

This kind of information is not “highlighted” as ABSTRACT section. Additionally, see comments at Pag 4-5, lines 89-91. There is some kind of redundancy in the general aims of the present manuscript.

Response: *As suggested by the reviewer in comments before, we have reworded the abstract to include the aims of this study. We have also reworded the sentence the reviewer refers to in this comment to make the reason why we are focusing on the extreme performers and their fecal microbiota.*

Line 147: The residual term distribution was not reported here.

Response: *As suggested, we have included the residual term distribution.*

Line 156: The residual term distribution was not reported here.

Response: *As suggested, we have included the residual term distribution.*

RESULTS/DISCUSSION

Both section are very well written and take into account the “essence” of this study. All information exploited in these sections are clear and very well evidenced for the readers.

Response: *Thank you for your comment.*
